# Role of the Drug Transporter ABCC3 in Breast Cancer Chemoresistance

**DOI:** 10.1371/journal.pone.0155013

**Published:** 2016-05-12

**Authors:** Sai A. Balaji, Nayanabhirama Udupa, Mallikarjuna Rao Chamallamudi, Vaijayanti Gupta, Annapoorni Rangarajan

**Affiliations:** 1 Department of Molecular Reproduction, Development and Genetics, Indian Institute of Science (IISc), Bangalore, 560012, India; 2 Department of Pharmacology, Manipal College of Pharmaceutical Sciences, Manipal University, Manipal, 576104, India; 3 Stand Life Sciences, Bangalore, 560024, India; Wayne State University, UNITED STATES

## Abstract

Increased expression of ABC-family of transporters is associated with chemotherapy failure. Although the drug transporters ABCG2, ABCB1 and ABCC1 have been majorly implicated in cancer drug resistance, recent studies have associated ABCC3 with multi drug resistance and poor clinical response. In this study, we have examined the expression of ABCC3 in breast cancers and studied its role in drug resistance and stemness of breast cancer cells in comparison with the more studied ABCC1. We observed that similar to ABCC1, the transcripts levels of ABCC3 was significantly high in breast cancers compared to adjacent normal tissue. Importantly, expression of both transporters was further increased in chemotherapy treated patient samples. Consistent with this, we observed that treatment of breast cancer cell lines with anti-cancer agents increased their mRNA levels of both ABCC1 and ABCC3. Further, similar to knockdown of ABCC1, knockdown of ABCC3 also significantly increased the retention of chemotherapeutic drugs in breast cancer cells and rendered them more chemo-sensitive. Interestingly, ABCC1 and ABCC3 knockdown cells also showed reduction in the expression of stemness genes, while ABCC3 knockdown additionally led to a reduction in the CD44^high^/CD24^low^ breast cancer stem-like subpopulation. Consistent with this, their ability to form primary tumours was compromised. Importantly, down-modulation of ABCC3 rendered these cells increasingly susceptible to doxorubicin in xenograft mice models *in vivo*. Thus, our study highlights the importance of ABCC3 transporters in drug resistance to chemotherapy in the context of breast cancer. Further, these results suggest that combinatorial inhibition of these transporters together with standard chemotherapy can reduce therapy-induced resistance in breast cancer.

## Introduction

Breast cancer is the most common solid tumour in women world over. According to WHO (World Health Organisation) guidelines, 14% of deaths are caused due to breast cancer every year. In India, breast cancer is one of the leading causes of cancer-related deaths among women. Breast cancer accounts for 25% to 32% of all female cancers in many Indian cities [1]. Even though various therapies have been designed against breast cancer, still treatment of breast cancer becomes a challenge due to acquisition of drug resistance and relapse of the tumour [2]. This study aims to understand the impediments in cancer chemotherapy and helps to identify approaches to reduce drug resistance in cancer.

Multi drug resistance (MDR) is a phenomenon that causes resistance to several kinds of drugs simultaneously. One of the main mechanisms of MDR is the overexpression of drug transporters called ATP binding cassette (ABC) transporters, also called as efflux pumps [3]. There are 48 members of this superfamily that have been categorised into 7 families as ABC A-G, and majorly 16 ABC transporters are involved in human diseases. Among these, ABCB1 (p-glycoprotein/MDR1), ABCC1 (MRP1) and ABCG2 (BCRP1) are the major drug transporters which have been widely implicated in drug resistance in several cancers. These transporters are found to be overexpressed in several cancers including lung, breast and pancreatic cancers, and their overexpression is inversely correlated with patient survival. Several reports, including work from our lab [4], have shown that treatment with chemotherapeutic drugs increases the expression of ABC transporters *in vitro*. Consistent with this, chemotherapy-treated patient samples have been shown to have elevated expression of ABC transporters. Indeed inhibition of ABC transporters leads to chemosensitization, suggesting that inhibitors of ABC transporters can improve treatment outcome.

Several members of the ABCC family have been implicated in drug resistance [5]. Upregulation of ABCC1 has been shown to be associated with more aggressiveness in hepatocellular carcinoma. Recent reports suggested that inhibition of ABCC1 with MK-571 reverses the doxorubicin resistance in H69AR small cell lung cancer cell line [6]. In pancreatic carcinoma, 5-flurouracil resistant capan1 cell line showed several folds upregulation of ABCC1 and ABCC3 expression compared to parental cell line [7]. Treatment with reversan, an ABCC1 inhibitor, improved tumour free-survival in mice [8]. ABCC3 was significantly expressed in hepatocellular carcinoma biopsies [9] and non-small cell lung carcinoma [10]. It served as a marker for multidrug resistance in non-small cell lung carcinoma and associated with poor survival. In another study, overexpression of ABCC3 in glioblastoma multiforme (GBM) was shown to be associated with high risk for death [11]. ABCC1 and ABCC3 may thus act as prognostic factors in many cancer subsets.

In breast cancers, ABCC1 expression has been correlated with patient survival following chemotherapy [12]. A tissue microarray analysis conducted on 281 breast cancer patients revealed that shorter disease free-survival was significantly associated with ABCC1 and ABCC11 expressions. It was also reported that these transporters were highly expressed in aggressive breast cancer subtypes [13]. Interestingly, ABCC5 expression in breast cancers has been associated with increased bone metastasis. ABCC3 is frequently amplified and overexpressed in HER2-positive breast cancer compared to HER2-negative breast cancer patient samples [14]. However, a functional relevance for ABCC3 expression in drug resistance and stemness aspect in breast cancers is not well established.

In this study, we examined the expression of the drug transporter ABCC3 in chemotherapy treated versus chemo-naive patient samples in comparison with that of ABCC1. We additionally investigated the functional consequences of overexpression of these transporters in drug retention, and the effects of their knockdown, using an RNA-i approach, in regulating drug sensitivity, breast cancer stemness, and tumor forming efficiency in immunocompromised mice.

## Materials and Methods

### Collection of normal, tumour and chemo-treated breast tissue samples

Adjacent normal (30 tissue samples), chemo-naive tumour breast tissues (66 tissue samples) and chemo-treated tumor tissue samples (n = 30) from grade III invasive breast ductal carcinoma were obtained from Kidwai Memorial Institute of Oncology (KMIO) Bangalore, in accordance with the Institutional Review Board and in compliance with the ethical guidelines of KMIO and the Indian Institute of Science (IISc). This study was approved by Institutional Human Ethics Committee, IISc. Patient consent was acquired in a written form before the surgery. The normal tissue was excised ~6 cm away from tumour and was confirmed by pathologists for absence of tumour cells. As per neo-adjuvant treatment protocol, patients were treated with 5-FU (5-flurouracil) and cyclophosphamide for 3 cycles (each cycle for 21 days). Surgically resected tumor tissue was collected and divided into two parts. One part harvested for RT-PCR and another part was used to isolate single cells for FACS based experiments. For RNA isolation, normal and tumour tissue chunks were collected in RNA later (Qiagen, Hilden, Germany). For FACS (Florescence activated cell sorting) analysis single cells were used. For experiments involving live culture, tissues were minced with DMEF-12 media containing collagenase and hylurinidase and minced tissue chunks were hybridized in 37°C for 12–14 hours, normal and tumour organoids were isolated by centrifugation. After 4 hours, organoids were trypsinized and single cells were isolated by passing through 100 μn cell strainer [15].

### Culture of normal and cancer cell lines

Immortalized breast cell line HBL-100 and breast cancer cell lines BT-474, MCF-7, MDA-MB-231, T-47D and HCC-1806 (obtained from ATCC) were cultured in DMEM (Sigma) supplemented with 10% FBS (Invitrogen), immortalized MCF-10A cells were cultured as described previously [4] in DMEM-F12 with growth factors (10 ng/ml hEGF, 1 mg/ml hydrocortisone, 10 mg/ml insulin, 4 ng/ml heparin) (Sigma Aldrich) and B27 (Invitrogen, Carlsbad, CA, USA) and 10% FBS. All media also included penicillin (1 kU/ml) and streptomycin (0.1 mg/ml), and patient derived cells were cultured in serum-free DMEM-F12 media supplemented with growth factors [16] and maintained under standard tissue culture conditions of 37°C in a humidified incubator.

### Transfections and stable cell generation

MDA-MB-231and BT-474 cells were transiently transfected with pCMV-ABCC1 expression (ABCC1 (OE)) or pCMV-ABCC3 expression (ABCC3 (OE)) constructs or pCMV-empty vector using lipofectamine-2000 (Invitrogen). After 48 hours of transfection cells were split into two portions: one portion was used for doxorubicin retention studies and another portion was used for RNA isolation. Expression constructs were kindly gifted by Dr. P Borst from Netherlands. ABCC1 and ABCC3 knockdown stable cells were generated by transfecting MDA-MB-231 cells and BT-474 cells with shRNA construct targeting ABCC1 (pGIPZ-ABCC1) or ABCC3 (pGIPZ-ABCC3) or non-targeting (pGIPZ-Non-targeting shRNA vectors; Thermo-scientific) using Lipofectamine-2000. Stable cells were generated by selection with puromycin (0.5 μg/ml) followed by flow cytometer based sorting (MoFlo-Beckman Coulter) for GFP expression (encoded by the vector) and were expanded and frozen for future use. Knockdown was confirmed by Real time PCR.

### Real Time PCR (qPCR)

Primary normal and tumour tissues (obtained from KMIO) were processed using motorized homogenizer, the snap frozen tissue (~100 mg) was ground and total RNA was isolated using Tri-reagent (Sigma Aldrich, St Louis, MO, USA) according to manufacturer’s protocol. cDNA was synthesized from 2 μg of total RNA using Gene-Amp RNA PCR cDNA synthesis kit (Applied Biosystems, Carlsbad, CA, USA). Primers were designed using Primer3 online tool. GAPDH and β2-microglobulin were used as normalizing controls. Sequence of primers used is provided in S1 Fig. Real Time PCR was performed according to manufacturer’s protocol using DyNAmo SYBR Green qPCR Kit (Finnzymes, Vantaa, Finland) with ROX as passive reference dye using Applied Biosystem’s 7900 HT Real Time PCR system. The following PCR program was used for all real time PCR based experiments: initial denaturation at 95°C for 5 minutes, followed by 40 cycles of denaturation at 95°C for 30 seconds, annealing at 60°C for 30 seconds, extension at 72°C for 30 seconds, and final extension at 72°C for 5 minutes.

### Real time PCR analysis strategy for patient samples

For quantification of gene expression, ct values of each gene was normalized to β2M and calibrated to the appropriate control sample using the SYBR Green-based comparative CT method (2-ΔΔCt). For comparison between normal and chemo-naive tumor tissues, ABCC1 and ABCC3 expression in tumor tissues was calculated using normal tissue sample expression as reference control. Further, ΔΔct of normal tissue samples was calculated by subtracting the average Δct of all the normal tissues (n = 30) from the individual Δct of normal tissue sample (n = 30). ΔΔct of chemo-naive tumor tissue samples was calculated by subtracting the average Δct of all the normal tissues (n = 30) from the individual Δct of tumor tissue sample (n = 66). For comparison between chemo-naïve tumor and chemotherapy treated samples, ABCC1 and ABCC3 expression of chemotherapy treated samples were calculated using chemo-naive (tumor) tissue sample expression as reference control. Further, ΔΔct of chemo-naive tissue samples was calculated by subtracting the average Δct of all the chemo-naive tissues (n = 66) from the individual Δct of chemo-naive tissue samples (n = 66). ΔΔct of chemo-therapy treated samples was calculated by subtracting the average Δct of all the chemo-naive tissue samples (n = 66) from the individual Δct of chemo-therapy treated tissue sample (n = 30). Fold change was calculated using the formula 2^-ΔΔct^ [17]

### Drug treatment strategies for real time PCR and retention studies

MDA-MB-231 and BT-474 cells were treated with 0.25 μM doxorubicin (DOX) or 0.2 μM of mitoxantrone (MXR) or 3 μM of 5-flurouracil (5-FU) (sigma Aldrich) for 4 days in incubator after 4 days cells were harvested for RNA isolation followed by cDNA preparation. For retention studies 1 μM of doxorubicin or 1 μM of mitoxantrone or 0.5 μM of rhodamine 123 was treated for only 1 hour.

### Drug retention assay

The cells were trypsinized, counted (1x10^5^) and incubated with doxorubicin {(1 μM) or rhodamine 123 {(0.5 μM) or mitoxantrone {(1 μM) in 37°C incubator for 60 minutes; vehicle control (Water) or unstained cells served as controls. Stained cells were washed twice with PBS and 10,000 events were analyzed per sample. Doxorubicin was measured using Exitation-488 nm and Emission-560 nm filters, while rhodamine and mitoxanthrone were measured using Exitation-511 nm and Emission-534 nm filters in BD FACS-Canto (BD Biosciences); Data was analysed using Summit 5.2 software.

### Cytotoxicity assay

Dose-dependent cytotoxic effects of doxorubicin, mitoxantrone and methotrexate (MTX) were measured by MTT assay as described earlier [18]. Briefly, cells were seeded (7x10^3^ cells/well in 100 μl) into 96 well plates in triplicates, after 12 hours of seeding, cells were treated with various concentrations of chemotherapeutic drugs for 48 hours. After 48 hours, 20 μl of 5 mg/ml of MTT reagent (Sigma Aldrich) was added into each well and incubated for 4 hours, then media was removed and 100 μl of DMSO was added to the wells and absorbance was measured at 575 nm using plate reader (Infinite M200 PRO-Tekan).

### CD44^high^/24^low^ analysis

CD44^high^/CD24^low^ assay was performed as described earlier (14). Briefly, MDA-MB-231 stable cells were trypsinized and counted, 1x10^5^ cells were taken from each condition and incubated at 37°C with 5% CO_2_ for 1 hour. Then CD44 antibody labelled with PE-CY-7 and CD24 antibody labelled with PE (BD Biosciences) were added and incubated in ice for 45 minutes with intermittent mixing. After incubation cells were washed twice with 1xPBS, and 10,000 events were analyzed per sample in BD-FACS canto-flow cytometer equipped with Exitation-488 nm and Emission-785 nm for PE-CY7 and Exitation-488 nm and Emission-578 nm filters for PE. Data was analysed using Summit 5.2 software.

### Annexin -V assay

For apoptosis analysis, MDA-MB-231 cells expressing NT, shABCC1 and shABCC3 cells (1x10^5^) were seeded into 12 well plate. Cell were treated with doxorubicin (1 μM) or methotrexate (30 μM) for 20 hours, trypsinized, counted and incubated with Annexin-V-PEcy 5.5 in Annexin-V binding buffer (BD-biosciences). After 15 minutes of incubation, cells were analyzed (10,000 events collected) using BD FACS canto equipped with (Exitation-488 nm and Emission-690 nm) filters. Data was analysed using Summit 5.2 software.

### *In vivo* tumour formation assay

Animal experiments were performed with approval from Institutional Animal Ethics Committee, IISc. Four-five week-old female NOD-SCID were used for *in vivo* animal experiments. The animals were housed under specific pathogen free conditions. Non-targeting shRNA cells were injected subcutaneously into left flank and ABCC3 knockdown cells were injected into right flank of each mouse. After 20 days of injection, when palpable bumps developed, the mice were randomized into 2 groups; one group was treated (tail vein) with vehicle control and another group with doxorubicin (2 mg/kg b.w) for every four days until 4 weeks. Tumour size was measured regularly. At the end of the experiments, the animals were sacrificed and tumours were dissected and tumor weight measured for size analysis. Tumor inhibition rate was calculated by Tumour Inhibition Rate (IR) formula, IR (%) = ((Wc−Wt)/Wc) × 100%, wherein Wc and Wt represent the mean tumor weight of the control group and treatment group.[19]

### Statistical analysis

Statistical significance determined using student’s t-test, ANOVA and two-way ANOVA. Curve-fit method was used to analyse IC_50_ value. Graph-pad prism software version 5 was used for all statistical tests and plotting the graphs. Results are shown as mean±SEM.

## Results

### ABCC3 is overexpressed in breast cancer samples and cancer cell lines

**Fig 1:**
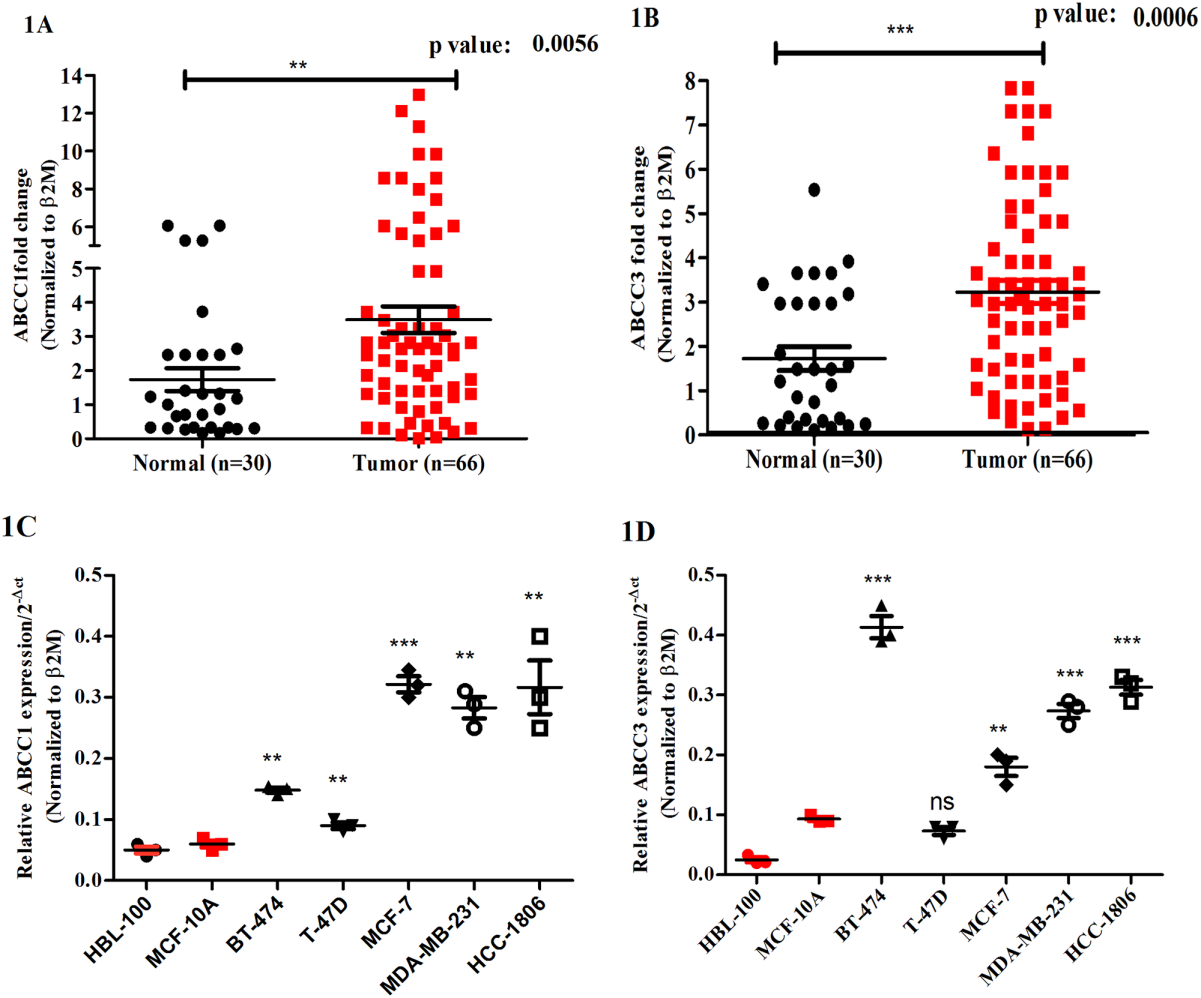
ABCC3 is overexpressed in breast cancer samples and cancer cell lines. A and B) ABCC1 and ABCC3 mRNA expression in primary normal breast tissues (n = 30) and primary grade III breast cancer tumors (n = 66) was determined by qPCR analysis. Relative quantification of ABCC1 (1A) and ABCC3 (1B) mRNA expression is standardized to β2M housekeeping gene and normalized to normal breast tissues. Error bar represent standard error of the mean (SEM); * = p<0.05, ** = p<0.01. C and D) Scatter plot shows ABCC1 (1C) and ABCC3 (1D) gene expression in immortalized breast cell lines (HBL-100 and MCF-10A) and breast cancer cell lines (BT-474, T-47D, MCF-7, MDA-MB-231 and HCC-1806) evaluated by qPCR. Error bar represent standard error of the mean (SEM); ** = p<0.01, *** = p<0.001, n = 3.

Although the expression of ABCC1 in breast cancers has been investigated by multiple studies [4, 20], the expression of ABCC3 has not yet been well investigated in grade III breast cancers. Hence, to begin to understand the role of these transporters in breast cancer progression, we first investigated the expression of ABCC3 in grade III invasive breast ductal carcinoma tissue samples and compared it with that of ABCC1 in the same samples. To do so, we performed real time quantitative PCR based study in patient samples. Our data revealed that ABCC1 was overexpressed (p = 0.0056) in breast cancer tissue compared to normal (Fig 1A, S1 and S2 Tables), consistent with previous studies [20]. In addition, we found a significant increase (p = 0.0006) in the expression of ABCC3 in breast cancer tissue compared to normal (Fig 1B, S3 and S4 Tables).

Further, we also investigated the expression of ABCC1 and ABCC3 in various breast cell lines ranging from immortalized to cancer cells. Interestingly we observed that most of the breast cancer cell lines tested (BT-474, MCF-7, T-47D, MDA-MB-231 and HCC-1806) showed significant upregulation of ABCC1 (Fig 1C) and ABCC3 (Fig 1D) compared to immortalized cell lines (HBL-100 and MCF-10A).

Together, our data is consistent with previous reports that have shown overexpression of ABCC1 in several cancers including breast cancers [21]. In addition, our data identifies increased expression of ABCC3 in grade III breast cancers in comparison to normal tissues.

### ABCC3 overexpression decreases the retention of anti-cancer agents

**Fig 2:**
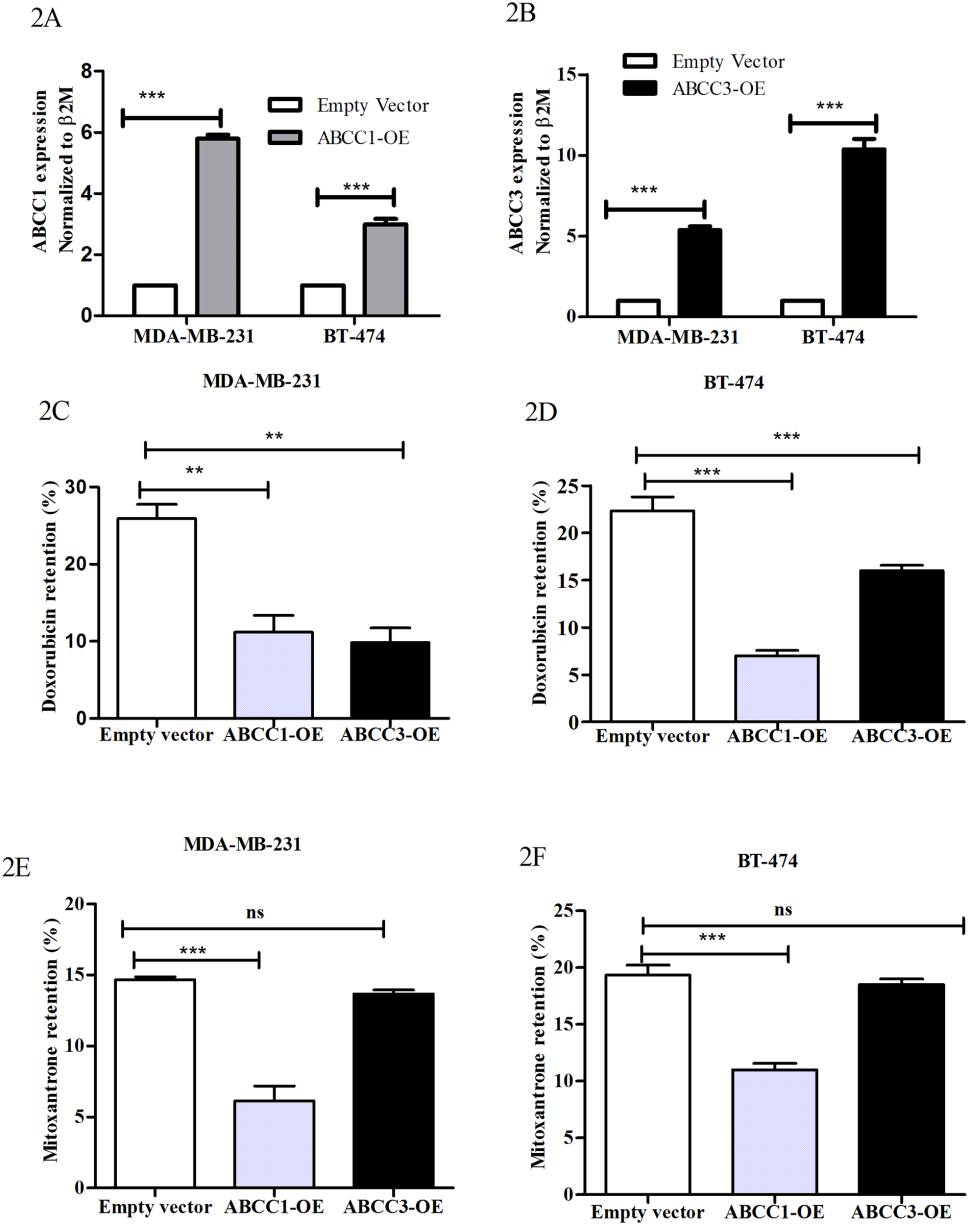
ABCC3 overexpression decreases the retention of anti-cancer agents. A and B) Bar graphs shows ABCC1 (2A) and ABCC3 (2B) gene expression in MDA-MB-231and BT-474 cells transiently transfected with pCMV (empty vector) or pCMV-ABCC1 overexpression vector (ABCC1 (OE)) or pCMV-ABCC3 overexpression vector (ABCC3 (OE)) of, evaluated by qPCR. Error bar represent standard error of the mean (SEM); *** = p<0.001, n = 3. C) Bar graphs shows the doxorubicin retention (%) in MDA-MB-231, transiently transfected with empty vector (MFI-18±1) or ABCC1 (OE), MFI-13±0.6 or ABCC3 (OE), MFI-13.5±1 and analyzed by flow cytometry. Error bar represent standard error of the mean (SEM); ** = p<0.01, *** = p<0.001, n = 3. D) Bar graphs shows the doxorubicin retention (%) in BT-474 transiently transfected with empty vector (MFI-20±1.2) or ABCC1 (OE). MFI-13.1±0.6 or ABCC3 (OE), 15±0.7 and analyzed by flow cytometry. Error bar represent standard error of the mean (SEM); ** = p<0.01, *** = p<0.001, n = 3. E) Bar graphs shows the mitoxantrone retention (%) in MDA-MB-231 transiently transfected with empty vector, MFI-19.6±0.2 or ABCC1 (OE), MFI-15.3±0.4 or ABCC3 (OE), MFI-18.1±0.67 and analyzed by flow cytometry. Error bar represent standard error of the mean (SEM); *** = p<0.001, n = 3. F) Bar graphs shows the mitoxantrone retention (%) in BT-474 transiently transfected with empty vector, MFI-21.6±0.4 or ABCC1 (OE), MFI-14.3±0.4 or ABCC3 (OE), MFI-19.1±0.97 and analyzed by flow cytometry. Error bar represent standard error of the mean (SEM); *** = p<0.001, n = 3.

Since increased expression of ABC transporters is associated with drug resistance primarily due to drug efflux, we investigated the direct effect of exogenous ABCC3 overexpression on chemotherapeutic drug retention, and compared it with that of ABCC1 expression, the well-studied family member. To do so we over expressed ABCC1 and ABCC3 genes by transient transfection of pCMV-ABCC1 and pCMV-ABCC3 constructs into MDA-MB-231 and BT-474 breast cancer cell lines. RT-PCR analyses revealed nearly 5-folds and 3-folds upregulation of ABCC1, while 5 folds and 12 folds upregulation of ABCC3, respectively in both the cells (Fig 2A and 2B). Further we observed that overexpression of ABCC1 significantly reduced the retention of doxorubicin and mitoxantrone in both MDA-MB-231 and BT-474 cells (Fig 2C–2F and S2A–S2D Fig). This is consistent with previous studies where collagen-1 treatment induced upregulation of ABCC1 was shown to lead to a reduction of doxorubicin retention in Jurkat and HSB2 leukemic T-cells [22]. Interestingly, overexpression of ABCC3 in these cells also significantly reduced the retention of doxorubicin (Fig 2C–2F), however retention of mitoxantrone was unaltered (Fig 2E and 2F). This is consistent with mitoxantrone not being an ABCC3 substrate (20). Taken together our results demonstrated that similar to ABCC1, overexpression of ABCC3 also reduces the retention of anti-cancer drugs in breast cancer cells.

### Chemotherapy increases ABCC3 gene expression

**Fig 3:**
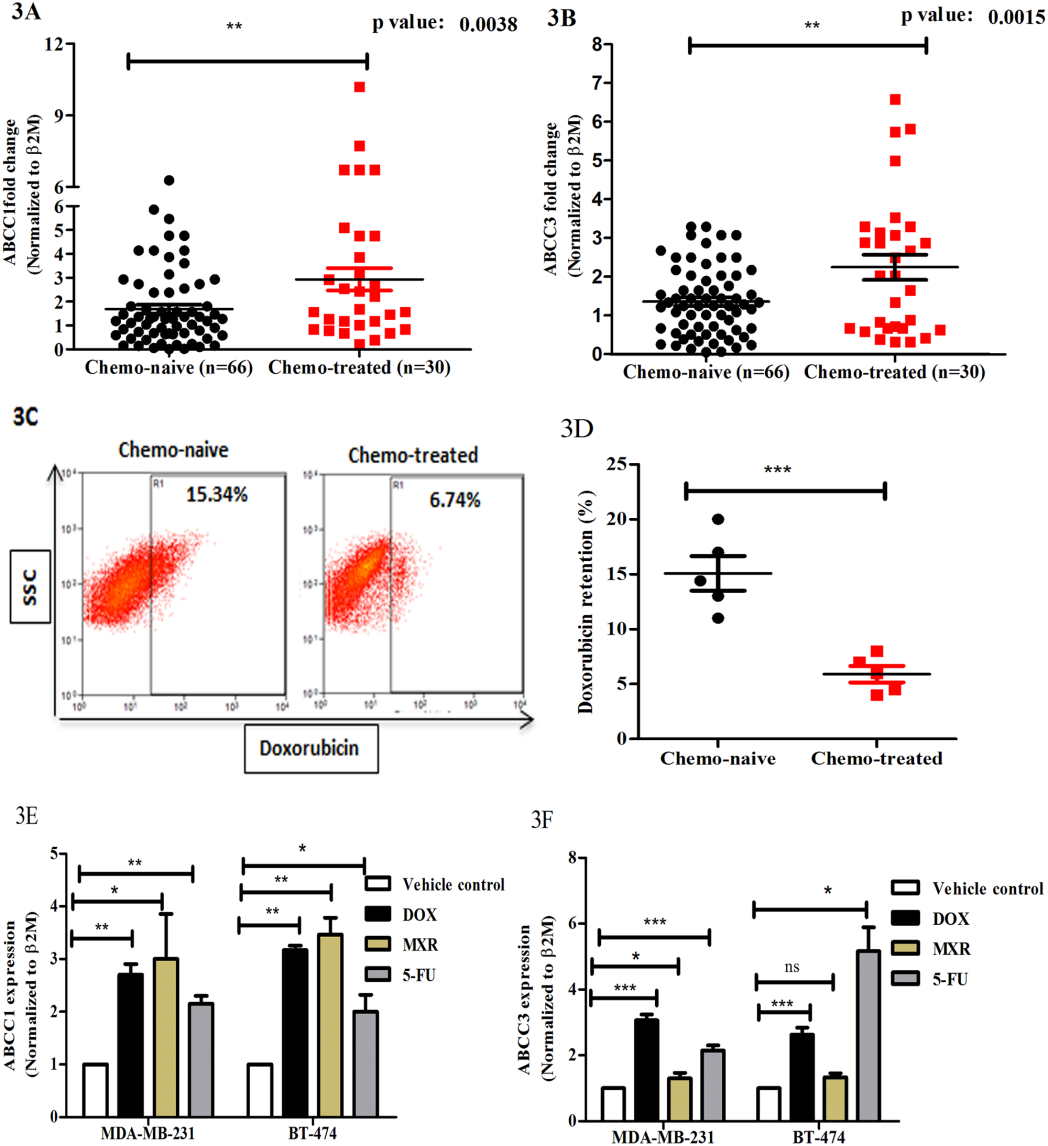
Chemotherapy increases ABCC3 gene expression. A and B) qPCR analysis of ABCC1 and ABCC3 expression in tumor (chemo-naive) tissues (n = 66) and chemo-therapy treated patient tissue samples (n = 30). Relative quantification of ABCC1 (3A) and ABCC3 (3B) mRNA expression was standardized to β2M housekeeping gene and normalized to chemo-naïve tumor tissue expression. Error bar represent standard error of the mean (SEM); * = p<0.05, ** = p<0.01. C and D) FACS plots (3C) shows the doxorubicin retention in chemo-naive tumour derived cells (n = 5, MFI-9.8±0.3) and CT treated tumour derived cells (n = 5, MFI-5.95±0.4). Bar graph (3D) shows the doxorubicin retention (%). Error bar represent standard error of the mean (SEM); ** = p<0.01, n = 3. E and F) Bar graph shows ABCC1 (3E) and ABCC3 (3F) gene expression in MDA-MB-231 and BT-474 cells treated with vehicle control, doxorubicin (0.25 μM), mitoxantrone (0.25 μM) and 5-FU (3 μM) evaluated by qPCR. Error bar represent standard error of the mean (SEM); * = p<0.05, ** = p<0.01, *** = p<0.001, n = 3.

Since chemotherapeutic treatment itself is known to upregulate the expression of ABC transporters [20], thus aiding drug resistance, we next investigated the effect of chemotherapy on the expression levels of ABCC3 using breast cancer patient-derived tissue samples. We first investigated the levels of ABCC1 that is known to be increased upon chemotherapy treatment [20], and would thus serve as a positive control. We performed real time PCR based analysis on freshly obtained chemotherapy treated breast cancer patient tissue samples (n = 30) and compared it with untreated (chemo-naive) patient sample (n = 66) set (the same set from Fig 1A and 1B). Our data revealed that chemotherapy-treated breast cancer samples showed higher expression of ABCC1 compared to chemo-naive samples (Fig 3A, S2 and S5 Tables), similar to previous report [20]. Additionally, our data revealed an increase in the expression of ABCC3 in chemotherapy-treated breast cancer patient samples (Fig 3B, S4 and S6 Tables) compared to chemo-naive samples, suggesting a possible role for ABCC3 in treatment-induced drug resistance in breast cancers.

To investigate if overexpression of ABCC1 and ABCC3 correlated functionally with drug resistance, we performed doxorubicin retention assays (n = 5) in primary cancer derived cells from the same chemotherapy treated and chemo-naive patient samples. Chemotherapy-treated samples showed significantly reduced retention of doxorubicin compared to chemo-naive samples (Fig 3C and 3D), suggesting that treatment with chemotherapy likely further induces drug resistance by decreasing the uptake of drugs.

Next we investigated the effects of chemotherapeutic drug treatment on the expression of ABCC1 and ABCC3 in established breast cancer cell lines. Our results revealed that upon 4 days of doxorubicin treatment (0.25 μM) ABCC1 and ABCC3 expression was upregulated in MDA-MB-231 and BT-474 cells compared to vehicle treatment (Fig 3E and 3F). Similarly, treatment with mitoxantrone (0.2 μM) for 4 days also increased ABCC1, but not ABCC3, expression compared to vehicle treatment. Since we observed that breast cancer samples derived from patients treated with 5-flurouracil and cyclophosphamide showed upregulation of ABCC1 and ABCC3, we further went ahead to check the expression of ABCC1 and ABCC3 upon 5-fluorouracil (3 μM) treatment in breast cancer cell lines. Interestingly we observed that ABCC1 and ABCC3 were upregulated upon 4 days of treatment with 5-flurouracil (Fig 3E and 3F). Together, our data revealed that treatment with chemotherapeutic drugs enhances the expression of ABCC1 and ABCC3 in breast cancer cells both in vivo and in vitro. While our data on ABCC1 are consistent with previous studies [4], we now additionally report the increased expression of ABCC3 on chemotherapy treatment in breast cancer cells.

### Knockdown of ABCC3 enhances doxorubicin retention in breast cancer cells

**Fig 4:**
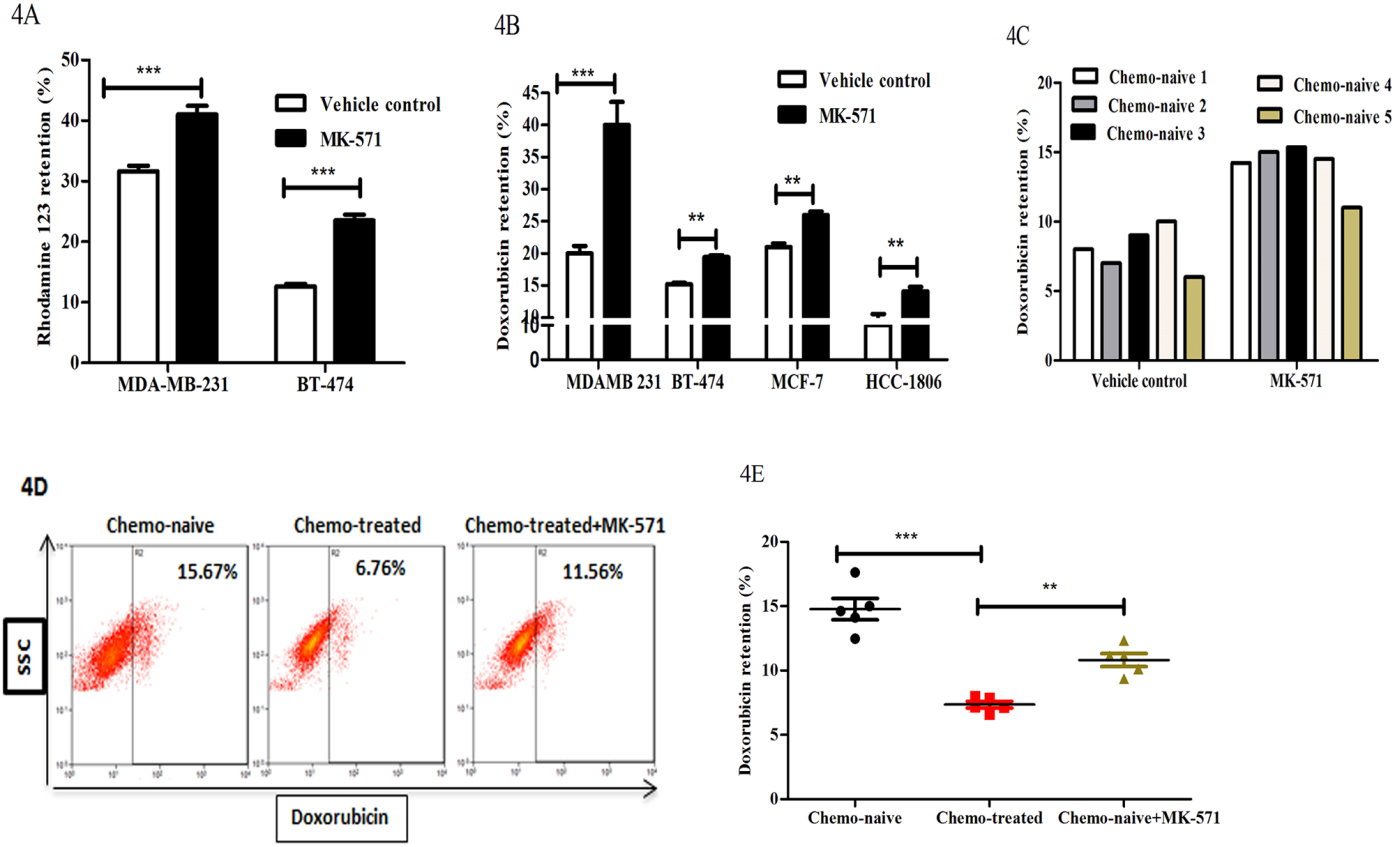
Knockdown of ABCC3 enhances doxorubicin retention in breast cancer cells. A) Bar graph shows the rhodamine-123 retention (%) in MK-571 treated MDA-MB-231 (MFI-341.1±4) and BT-474 cells (324.1±0.6) and vehicle treated MDA-MB-231(MFI-302±2) and BT-474 cells (295±5) respectively. Error bar represent standard error of the mean (SEM); ** = p<0.01, *** = p<0.001, n = 3. B) Bar graphs shows the doxorubicin retention (%) in MK-571 treated MD-MB-231 (MFI-11±0.32), BT-474 (10.5±0.6), MCF-7 (MF-13.2±0.3) and HCC-1806 (MFI-5.9±0.5) and vehicle treated MD-MB-231 (MFI-15.5±0.3), BT-474 (8.3±0.9), MCF-7 (12.1±0.2) and HCC-1806 (MFI-4.1±0.2) breast cancer cell lines respectively. C) Bar graphs shows the doxorubicin retention (%) in MK-571 treated chemo-naive tumour derived cells (MFI-14.5±0.8) compared to vehicle treated cells (MFI-9.7±0.5). Error bar represent standard error of the mean (SEM); ** = p<0.01, *** = p<0.001, n = 3. D and E) FACS plots (4D) shows the doxorubicin retention in chemo-naive tumour derived cells (n = 5, MFI-9.8±0.45), CT treated tumour derived cells (n = 5, MFI-6.1±0.3) and CT treated tumour derived cells treated with MK-571 (MFI-7.7±0.4). Scatter plot shows the doxorubicin (%) retention in tumour derived cells (4E). Error bar represent standard error of the mean (SEM); n = 3.

To further corroborate the expression of ABCC3 with drug transport and chemoresistance, we investigated the effect of inhibition/knockdown of these transporters. To do so, we first standardized drug retention assays in the presence of pharmacological inhibitors of ABC transporters. Since such inhibitors are currently available only for ABCC1, we proceeded to check the effect of pharmacological inhibition of ABCC1, which would additionally serve as positive control. Here we pre-treated breast cancer cell lines (MDA-MB-231 and BT-474) with 20 μM of MK-571, a specific ABCC1 inhibitor, for 1 hour, followed by treatment with 0.5 μM rhodamine 123, a standard substrate for retention studies [23]. We observed significant increase in the retention of rhodamine 123 upon ABCC1 inhibition compared to vehicle treated (DMSO) cells (Fig 4A and S3A Fig). We next performed doxorubicin retention assay upon ABCC1 inhibition to check the retention of doxorubicin in various breast cancer cell lines (MDA-MB-231, BT-474, MCF-7 and HCC-1806) as well as in primary breast cancer tissue samples. We observed that doxorubicin retention was also increased in MK-571 treated cells compared to vehicle treated cells (Fig 4B and S3B Fig). Similar results were obtained with primary breast cancer tissue-derived cells (Fig 4C and S3C Fig). We next asked if ABCC1 was involved in the observed (Fig 3C and 3D) reduction in doxorubicin retention in the chemotherapy treated patient samples. To address this, we investigated the effects of MK-571 treatment in drug retention by chemotherapy treated patient sample derived cells. We had previously observed that doxorubicin retention was 2 folds lower in chemotherapy treated patient samples compared to chemo-naive patient samples. Interestingly, we observed that inhibition of ABCC1 with MK-571 in chemotherapy treated patient samples increased their doxorubicin retention (Fig 4D and 4E). Thus, our data revealed that increase in the expression of ABCC1 on chemotherapy treatment reduces the retention of doxorubicin in breast cancer cells and this could be rescued by MK-571.

**Fig 5:**
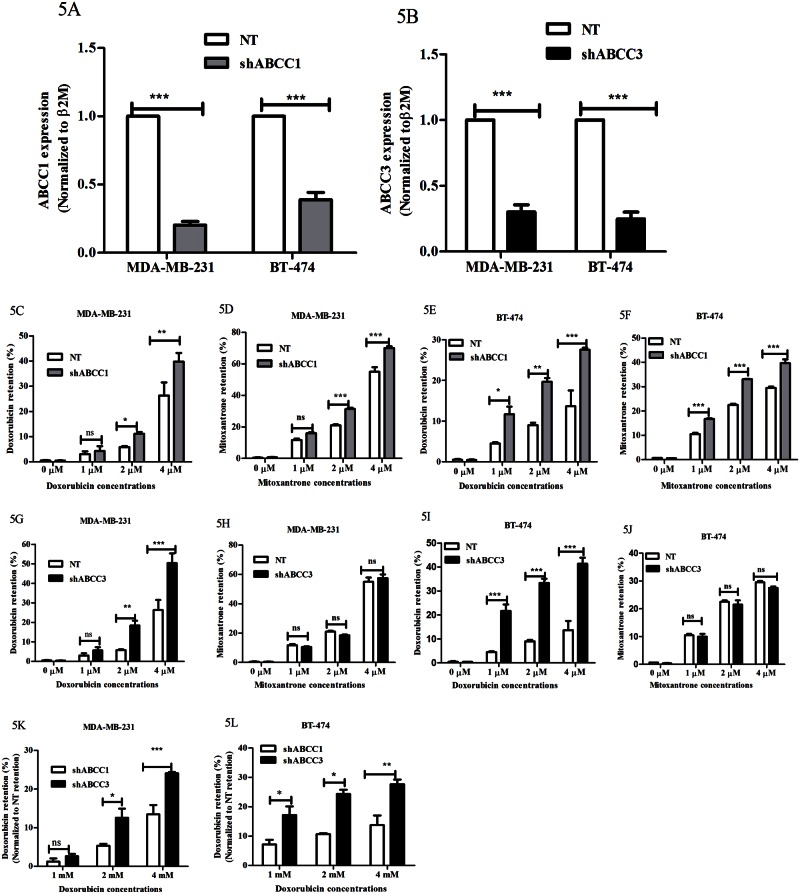
Knockdown of ABCC3 enhances doxorubicin retention in breast cancer cells. A and B) Bar graph shows ABCC1 (5A) and ABCC3 (5B) gene expression in MDA-MB-231 and BT-74 cells stably expressing non-targeting shRNA (NT), shABCC1 or shABCC3 shRNA evaluated by qPCR. Error bar represent standard error of the mean (SEM); *** = p<0.001, n = 3. C, D, E and F) Bar graphs shows the drug retention (%) in MDA-MB-231 and BT-474 cells stably expressing NT or shABCC1 treated with increasing concentrations of doxorubicin or mitoxantrone. Error bar represent standard error of the mean (SEM); ** = p<0.01, *** = p<0.001, n = 3. G, H, I and J) Bar graphs shows the drug retention (%) in MDA-MB-231 and BT-474 cells stably expressing NT or shABCC3 treated with increasing concentrations of doxorubicin or mitoxantrone. Error bar represent standard error of the mean (SEM); ** = p<0.01, *** = p<0.001, n = 3. K and L) Bar graphs show percentage drug retention in MDA-MB-231 and BT-474 cells stably expressing shABCC1 or shABCC3 and treated with increasing concentrations of doxorubicin. Error bar represent standard error of the mean (SEM); * = p<0.05, ** = p<0.01, *** = p<0.001, n = 3.

We next proceeded to test the effects of ABCC3 inhibition on drug retention in breast cancer cells. Since specific pharmacological inhibitors for ABCC3 are not currently available, we have undertaken shRNA mediated knockdown approach. To do so, we generated stable knockdowns of ABCC3 in MDA-MB-231 and BT-474 cells by transfecting pGIPZ-ABCC3 shRNA and compared it with the effect of ABCC1 knockdown using pGIPZ-ABCC1 shRNA that served as positive control. Stable cell lines expressing non-targeting shRNA (NT) were used as control. A qPCR analysis revealed greater than 60% knockdown of ABCC1 and ABCC3 in both the cell lines by real time PCR (Fig 5A and 5B). We first performed doxorubicin retention assays and observed that knockdown of ABCC1 in both MDA-MB-231 and BT-474 cells resulted in increased doxorubicin retention at two different concentrations of doxorubicin 2 μM and 4 μM (Fig 5C, 5E and S4A Fig). Similar results were obtained with yet another anti-cancer drug mitoxantrone (Fig 5D and 5F). Thus, our these data revealed that similar to inhibition of ABCC1 with MK-571, knockdown of ABCC1 in breast cancer cells also promoted their increased retention of doxorubicin, consistent with a previous report on Jurkat cells [22].

We next proceeded to test the effects of ABCC3 knockdown. We observed that doxorubicin retention was significantly upregulated in ABCC3 knockdown cells (Fig 5G and 5I), whereas mitoxantrone retention was similar to non-targeting shRNA carrying cells (Fig 5H and 5J), consistent with the fact that mitoxantrone is not a substrate for ABCC3. Thus, our data revealed that similar to ABCC1, ABCC3 knockdown also leads to increased retention of anticancer drugs (Fig 5G–5J), suggesting a possible causal role for ABCC3 in treatment-induced drug resistance. Interestingly, we found that doxorubicin retention was significantly increased upon ABCC3 knockdown compared to ABCC1 knockdown cells (Fig 5K and 5L). Together, these data suggested that knockdown or inhibition of specific ABC transporters that are induced in response to chemotherapy treatment might improve the effect of anticancer drugs by increasing drug retention.

### Knockdown of ABCC3 improves sensitivity to chemotherapeutic agents

**Fig 6:**
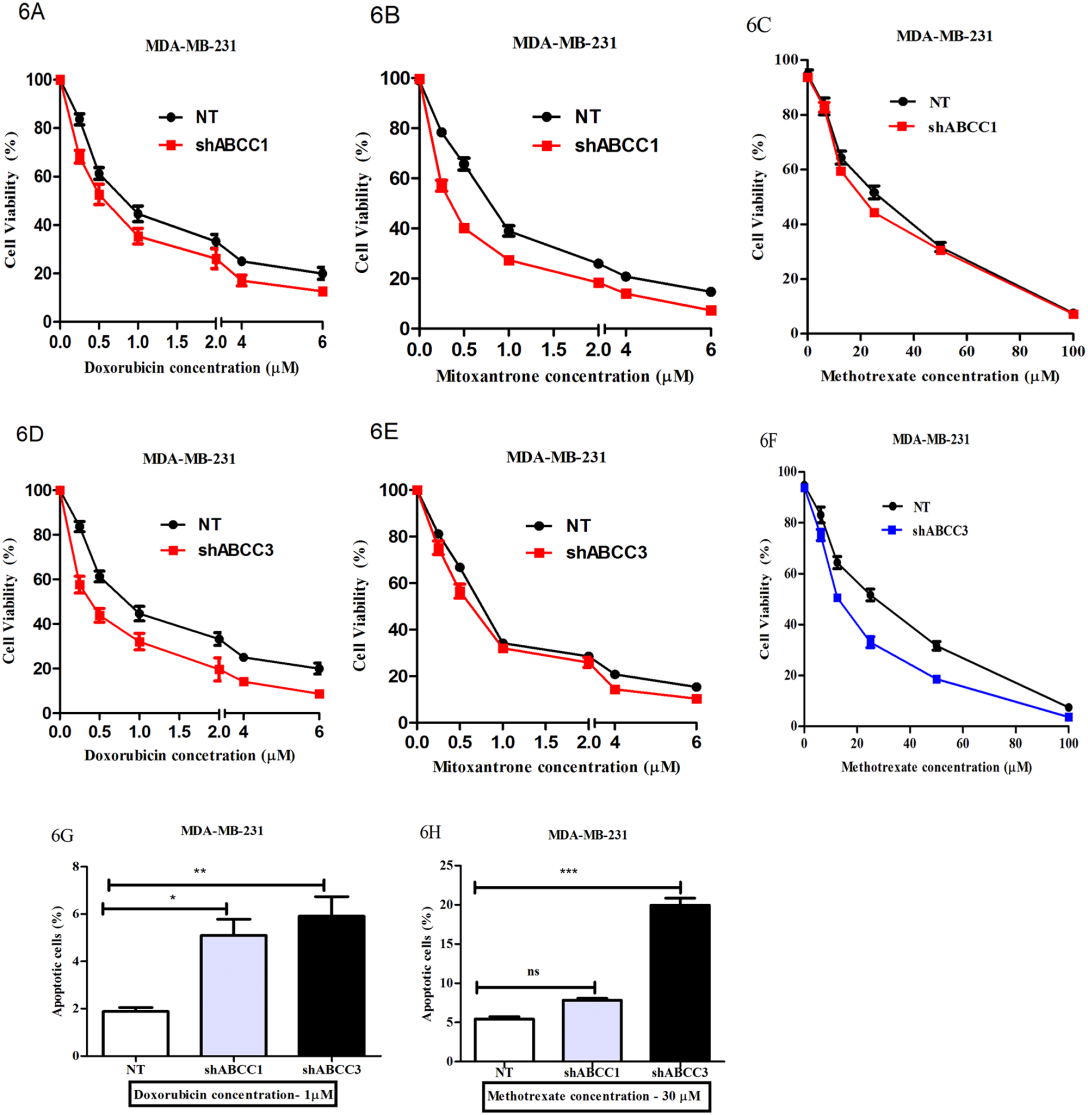
Knockdown of ABCC3 improves sensitivity to chemotherapeutic agents. A-F) The line graph shows the cell viability (%) in MDA-MB-231 cells stably expressing NT or shABCC1 (6A, 6B and 6C) or shABCC3 (6D, 6E and 6F) treated with increasing concentrations of doxorubicin, mitoxantrone and methotrexate for 48 hours; Error bar represent standard error of the mean (SEM); n = 3. G and H) The bar graph shows the percentage of apoptotic cells upon doxorubicin (6G) or methotrexate treatment (6H) in MDA-MB-231 cells stably expressing NT, shABCC1 and shABCC3 evaluated by Annexin V assay; Error bar represent standard error of the mean (SEM); * = p<0.05, ** = p<0.01, *** = p<0.001, n = 3.

Since we observed that knockdown of ABCC3 led to increased doxorubicin retention, we next gauged the effect of its knockdown on chemosensitivity, and compared it with experiments on ABCC1 knockdown that served as positive control. As expected, knockdown of ABCC1 led to a reduction in IC_50_ for doxorubicin from 0.98 μM in NT cells to 0.60 μM (Fig 6A and S7 Table). Similarly we investigated the IC_50_ value for mitoxantrone and methotrexate. Here we observed that IC_50_ value for mitoxantrone was reduced from 0.81 μM (NT) to 0.36 μM upon ABCC1 knockdown (Fig 6B and S7 Table). However, IC_50_ value for methotrexate did not change much upon ABCC1 knockdown (Fig 6C and S7 Table). This is again consistent with methotrexate not being a good substrate for ABCC1. Thus, knockdown of ABCC1 rendered breast cancer cells chemo-sensitive. This is consistent with previous results wherein IC_50_ value of etoposide was decreased upon knockdown of ABCC1 in MCF-7 cells [8].

To begin to understand the functional role of ABCC3 in chemoresistance, we performed same experiments using MDA-MB-231 stably expressing shABCC3 cells. In these cells, we observed that the IC_50_ values for doxorubicin and methotrexate reduced from 0.98 μM to 0.41 μM and from 24.12 μM to 13.35 μM, respectively (Fig 6D, 6F and S7 Table). However, the IC_50_ for mitoxanthrone was only slightly reduced from 0.81 μM to 0.62 μM (Fig 6E and S7 Table), consistent with lack of mitoxanthrone retention in the ABCC3 knockdown cells as observed previously (Fig 5H). Thus, our data showed that consistent with increased doxorubicin retention, knockdown of ABCC3 also reduces the IC_50_ for chemotherapeutic drugs, much more than the ABCC1 knockdown, suggesting that inhibition or preventing the upregulation of ABCC3 might be efficacious for breast cancer treatment.

Further we investigated the chemotherapeutic effects on ABCC1 and ABCC3 knockdown cells using apoptosis assay. Here first we treated MDA-MB-231 cells expressing shABCC1 or shABCC3 or NT cells with either doxorubicin (1 μM) or methotrexate (30 μM) for 20 hours, and then we observed the percentage of apoptosis using Annexin V assay. We observed that percentage of apoptosis increased upon doxorubicin treatment in ABCC1 and ABCC3 knockdown cells compared to control NT cells (Fig 6G and S5A Fig), but in methotrexate treatment apoptosis percentage was increased only upon ABCC3 knock-down but not in ABCC1 knock-down (Fig 6H and S5A Fig), this is consistent with the reduction in methotrexate IC_50_ value only in ABCC3 knock-down cells (S7 Table). Thus, our data revealed that similar to knockdown of ABCC1, ABCC3 knockdown also increased the drug-sensitivity of breast cancer cells. Our data further revealed a novel observation that identifies the knockdown of ABCC3, but not ABCC1, sensitizes breast cancer cells to methotrexate, a routinely used drug to treat breast cancers, together highlighting an important role for ABCC3 in breast cancer drug resistance.

### Knockdown of ABCC3 reduces stemness

**Fig 7:**
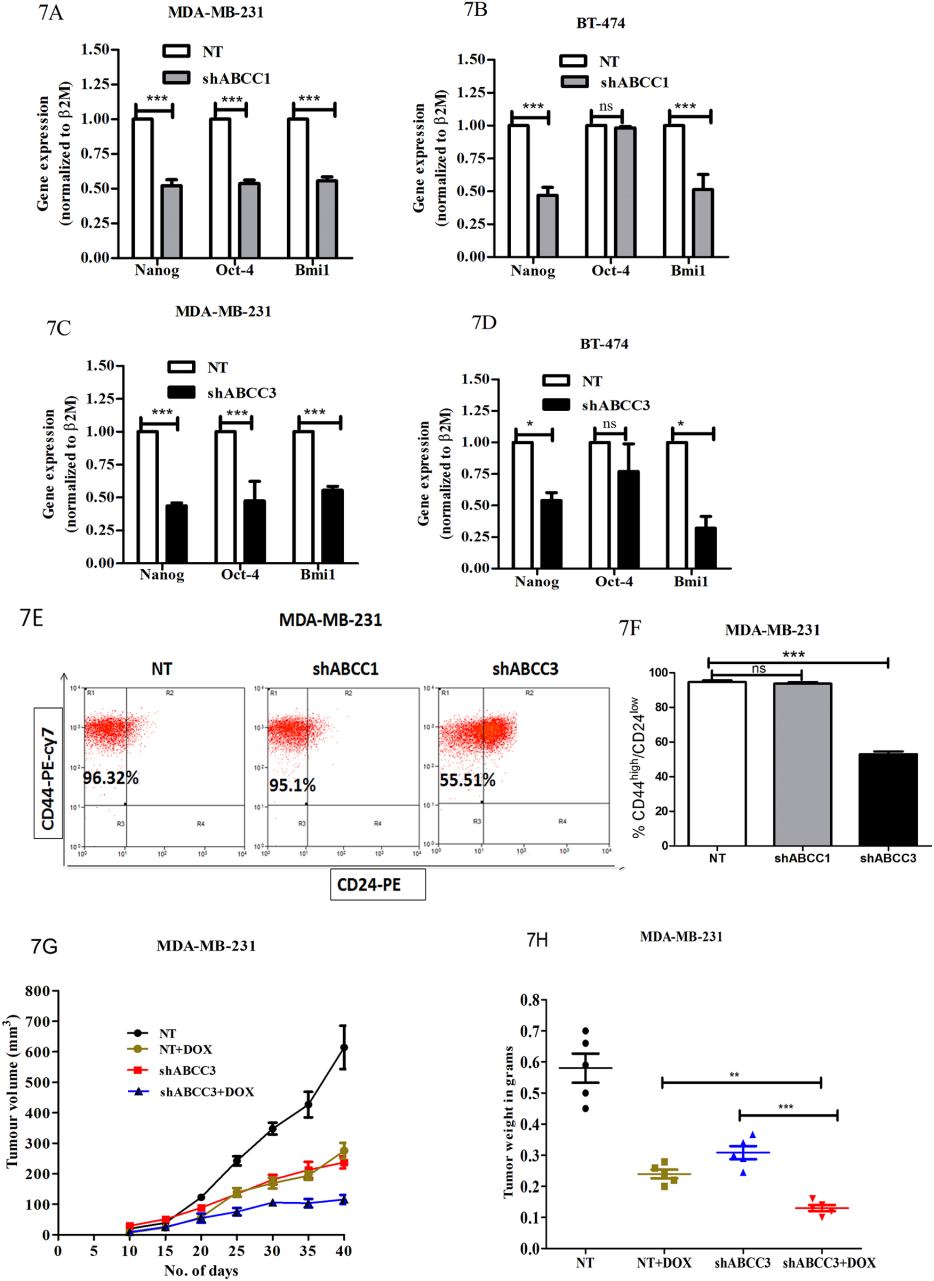
Knockdown of ABCC3 reduces stemness. A-D) Bar graphs show gene expression of Nanog, Oct4 and Bmi1 in MDA-MB-231 and BT-474 cells stably expressing NT, shABCC1 (7A and 7B) and shABCC3 (7C and 7D) evaluated by qPCR; Error bar represent standard error of the mean (SEM); * = p<0.05, *** = p<0.001, n = 3. E and F) FACS plot shows the CD44^high^/CD24^low^ expression in MDA-MB-231 cells stably expressing NT, shABCC1 and shABCC3 shRNA (7E). Bar graph shows the percentage of CD44^high^/24^low^ expression (7F). Error bar represent standard error of the mean (SEM); *** = p<0.001, n = 3. G and H) The line graph shows tumor growth kinetics of MDA-MB-231 cells expressing NT or shABCC3 and injected subcutaneously (1.5x10^6^ cells/injection) into 5 weeks NOD-SCID mice. Doxorubicin treatment was given 20 days after injection and continued for 4 weeks (7G). Tumour growth was monitored for the indicated period. At the End of the treatment tumours were isolated and tumor weight was measured (7H). Error bar represent standard error of the mean (SEM); ** = p<0.01, *** = p<0.001, n = 5.

Since drug resistance is correlated with cancer cell stemness [24], and a direct role for ABC transporters in the regulation of cancer cell stemness is not yet well explored, we next investigated the effects ABCC3 knockdown on stemness gene expression using real time PCR based approach, and compared it with that of ABCC1 that has been implicated in cancer stemness. We observed that upon ABCC1 knockdown stemness genes like Nanog and Bmi1 were down regulated in MDA-MB-231 (Fig 7A) and BT-474 cell-lines (Fig 7B). However, Oct-4, yet another stemness gene, was down regulated only in MDA-MB-231 cells but not in BT-474 cells. Interestingly similar results were obtained with ABCC3 knockdown cells in both MDA-MB-231 (Fig 7C) and BT-474 cells (Fig 7D). Thus, knockdown of ABCC3, similar to that of ABCC1, led to a decrease in the expression of stemness related genes. We further compared the effect of ABCC1 and ABCC3 knockdown on CD44^high^/24^low^ marker profile that identifies breast cancer stem cells [25]. We found that (CD44^high^/24^low^) stem like cell population was significantly reduced upon ABCC3 knockdown (Fig 7E and 7F) in MDA-MB-231 cells. However, knockdown of ABCC1 failed to show this effect (Fig 7E and 7F). Thus, we show that knockdown of ABCC1 and ABCC3 leads to reduction of stemness gene expression, and, importantly, that knockdown of ABCC3 further showed reduced breast cancer stem-like cell population.

To further corroborate our results with drug resistance *in vivo*, we tested the effects of doxorubicin on xenograft tumours generated in immunocompromised mice. Since knockdown of ABCC3 led to a significant retention of doxorubicin, and also decreased the breast cancer stem-like cell population, we went ahead and undertook in vivo experiments with ABCC3 knockdown cells. Consistent with a reduction in the number of stem-like cancer cells, we observed that knockdown of ABCC3 led to reduced tumor growth compared to control NT cells, which was further reduced on doxorubicin treatment (Fig 7G, 7H and S8 Table). The combined effect of ABCC3 depletion and doxorubicin treatment led to greater tumor growth retardation than individual treatments (Fig 7G, 7H and S8 Table).

## Discussion

Despite advances in cancer treatment strategies, the survival rate of patients with breast cancer is still low because of development of multi drug resistance. Multi drug resistance in breast cancer has been majorly attributed to efflux of drugs by ABC transporters. Recent reports suggest that overexpression of ABCB1 leads to increase in the gene expression of CD44 in breast cancer cells [26] Therefore, understanding the expression and functional significance of specific ABC transporters will help in the development of novel treatment strategies aimed at targeting or reducing their expression in order to achieve better treatment response. In this study we investigated the expression of ABCC1 and ABCC3 in breast cancers and assessed their role in cancer drug resistance and stemness. We observed that similar to ABCC1, the expression of ABCC3 is elevated in breast cancers, particularly following chemotherapeutic drug treatment. Similar to ABCC1, overexpression of ABCC3 also led to decreased drug retention, while its knockdown increased drug retention and increased chemosensitivity. These data suggested that similar to ABCC1 [27], ABCC3 could be both a prognostic indicator and a therapeutic target in breast cancer treatment.

Expression of ABCC1 has been shown to be significantly increased in neuroblastomas and its overexpression is associated with poor patient survival. In non-small cell lung cancer ABCC3 was shown to be highly expressed and it served as a drug resistance marker [28]. In this study we show that ABCC1 and ABCC3 were significantly overexpressed in a large number of breast cancer samples compared to normal. Similar trend was observed in several breast cancer cell lines compared to immortalized cells. Our data is consistent with previous reports on overexpression of ABCC1 in breast cancers [12], while additionally identifying ABCC3 overexpression in high grade breast cancers.

Chemotherapy leads to increased expression of several ABC transporters in multiple cancer types [29]. Consistent with this we observed that the expressions of ABCC1 and ABCC3 in chemotherapy-treated breast cancer derived tissue samples were significantly upregulated compared to chemo-naive breast cancer derived tissue samples. In addition, we observed that treatment of breast cancer cell lines *in vitro* with anti-cancer drugs routinely used in the clinic to treat breast cancers (doxorubicin, mitoxantrone and 5-flurouracil) also increased the expression of ABCC1 and ABCC3 in these cells. Further our data on drug retention analysis showed that the cells derived from chemotherapy treated samples had lower retention of doxorubicin compared to the cells derived from chemo-naive samples. Together, these data suggest that preventing the treatment-induced upregulation of ABC transporters might improve the efficacy of chemotherapy.

Several mechanisms have been proposed for the chemotherapeutic drug-induced upregulation of ABC transporters [30]. Adriamycin-induced upregulation of Twist has been shown to increase the expression of ABCB1 [31]. Consistent with this, in our previous study we have identified EMT-transcription factor binding sites on the promoters of several ABC transporters, and showed chemotherapy induces increase in several EMT factors as a possible mechanism for drug-induced upregulation of ABC transporters [4]. In addition, several studies have suggested epigenetic regulation of ABC transporter expression in response to chemotherapeutic drugs. For example, promoter demethylation leading to upregulation of ABCB1 has been observed in doxorubicin selected CCRF-CEM cells (CEM-A7R) [32]. Similar results were obtained in vincristine-selected KB3-1 cells [33]. Recent reports have shown that the chromatin-remodelling proteins have an impact on ABCG2 expression in several carcinoma cell lines [34, 35]. In another study it was shown that epigenetic up-regulation of ABCG2 is an upstream event leading to ABCG2-mediated MDR through chemotherapeutic drug-induction [30]. Thus, better understanding of the mechanisms that promote upregulation of ABC transporters upon chemotherapy can help identify means to prevent this.

Inhibition or downmodulation of some ABC transporters has been shown to increase the accumulation of drugs and promote cell death [22]. Inhibition of P-gP (ABCB1) in MDA-MB-435S cells showed increased retention of doxorubicin. It was also shown that inhibition of ABCC1 leads to increased accumulation of doxorubicin in T-lymphocyte cells [21]. In addition to the above, we have shown that inhibition or knockdown of ABCC1 increases the accumulation of doxorubicin and mitoxantrone in two different breast cancer cell lines (MDA-MB-231 and BT-474). Also, our data showed that knockdown of ABCC3, significantly increased the retention of doxorubicin in breast cancer cell lines, better than that achieved by ABCC1 knockdown. Consistent with this, we noticed that the knockdown of ABCC3 led to higher reduction in the IC_50_ values for doxorubicin (S7 Table). Whether such reductions have therapeutic significance remains to be established. One possibility is that only a specific sub-population is rendered more sensitive, and if this happens to be the drug resistant subpopulation, then even slight reductions in IC_50_ value may be therapeutically significant.

Further Annexin V assays revealed that doxorubicin and methotrexate treatment of ABCC1 and ABCC3 knockdown cells showed 5 to 6 fold increased apoptosis compared to NT cells. These results suggest that knockdown of ABCC3, similar to knockdown of ABCC1, can improve the sensitivity of breast cancer cells to chemotherapeutic drugs. Thus, our study reveals that, knockdown of ABCC3 significantly increases drug retention, chemosensitivity and apoptosis in breast cancer cells.

Further we investigated other cancer-related biological phenomena that could be modulated by these transporters. It has been reported that knockdown of ABCC1 and ABCC4 decreases sphere formation efficiency of neuroblastoma cells [36], suggestive of decreased stemness. Interestingly we observed the reduction of stemness genes expressions (like Nanog and Bmi1) upon ABCC1 and ABCC3 knockdown in MDA-MB-231 and BT-474 cell lines. Further, CD44^high^/CD24^low^ fraction, which has been identified as the stem-like cells in breast cancer, was also reduced upon knockdown of ABCC3, but not by ABCC1 knockdown. These results indicated that these transporters might also be involved in the regulation of cancer stemness; however the mechanisms by which inhibition or knockdown of ABC transporters might contribute to cancer stemness remains to be investigated. Knockdown of various ABC transporters have indeed been shown to affect different cellular processes. For example, knockdown of ABCG2 has been shown to reduce the expression of Nanog through p53 regulation in Embryonic stem cells [37]. In lung cancer cell lines it was shown that ABCG2 has a typical role in the regulation of cell proliferation. Knockdown of ABCG2 led to G0/G1 cell cycle arrest associated with up-regulation of p21 and downregulation of cyclin D3 [38]. In another study it was reported that mice lacking ABCC1 are viable and fertile but have a defect in inflammatory response due to decreased secretion of LTC4, which is an endogenous substrate of ABCC1 [39, 40]. However the precise mechanisms that lead to decrease in stemness upon inhibition/knockdown of specific ABC transporters remain to be investigated.

Amongst the two transporters, knockdown of ABCC3 led to a greater increase in the retention of doxorubicin and a higher reduction in IC_50_ value compared to the knockdown of ABCC1. This prompted us to study the effect of this particular transporter in *in vivo* mouse model. Consistent with a reduction in the stem-like cell population in vitro, knockdown of ABCC3 reduced the tumor formation ability *in-vivo*. Treatment with doxorubicin further increased growth retardation, suggesting that a combination of ABCC3 inhibition together with doxorubicin will likely lead to better therapeutic end point. Thus this study has highlighted the importance of ABCC3 in breast cancers and further suggests that targeting this transporter might help to alleviate therapy-induced resistance.

## Conclusion

Thus, our study revealed that similar to ABCC1, ABCC3 is also overexpressed in grade III primary breast cancers. Importantly, *in vivo* and *in vitro* drug treatment led to a further increase in their expression levels, indicating their possible role in therapy-induced resistance in breast cancer cells. Consistent with this, overexpression of ABCC3 correlated with decreased drug retention, while knockdown of ABCC3 increased drug retention and apoptosis. Taken together, these results suggest that treatment with anticancer agents in combination with inhibition/down modulation of ABCC3 may be an effective clinical strategy for treating breast cancers. Further, our study highlights a role for ABCC3 in chemotherapy induced drug resistance and in the regulation of cancer stemness in the context of breast cancer.

## Supporting Information

S1 Fig Primers for Real time PCR experiments. This data file contains a list of the primers and sequences for the same, that were used in Real time PCR experiments.
(PDF)

S2 Fig Effect of ABCC3 overexpression on chemotherapeutic drug retention. FACS plots representing the drug retention in MD-AMB-231 and BT-474 cells (S2B and S2D) transiently transfected with empty vector or ABCC1 (OE) or ABCC3 (OE); n = 3.
(PDF)

S3 Fig Effect of MK-571 on Rhodamine and Doxorubicin retention in breast cancer cells. FACS plots represents rhodamine-123 retention in MK-571 treated breast cancer cells (S3A), doxorubicin retention in MK-571 treated breast cancer cells (S3B) and in primary breast cancer cells (S3C). n = 3.
(PDF)

S4 Fig Effect of ABCC3 knockdown on doxorubicin retention. FACS plots shows retention of increasing concentrations of doxorubicin in MDA-MB-231 cells expressing NT or shABCC1 or shABCC3.; n = 3.
(PDF)

S5 Fig Effect of ABCC3 knockdown on drug induced apoptosis. The FACS plots representing the percentage of Annexin-V-PE-cy5 staining in MDA-MB-231 expressing NT, shABCC1 and shABCC3 cells treated with doxorubicin or methotrexate for 20 hours.
(PDF)

S1 Table Expression of ABCC1 in normal breast tissue samples. Represents the qPCR results of ABCC1 expression in normal breast tissue samples.
(XLSX)

S2 Table Expression of ABCC1 in tumor breast (Chemo-naive) tissue samples. Represents the qPCR results of ABCC1 expression in tumor breast (chemo-naive) tissue samples.
(XLSX)

S3 Table Expression of ABCC3 in normal breast tissue samples. Represents the qPCR results of ABCC3 expression in normal breast tissue samples.
(XLSX)

S4 Table Expression of ABCC3 in tumor breast (Chemo-naive) tissue samples. Represents the qPCR results of ABCC3 expression in tumor breast tissue (chemo-naive) samples.
(XLSX)

S5 Table Expression of ABCC1 in chemotherapy treated tumor samples. Represents the qPCR results of ABCC1 expression in tumor breast tissue (chemotherapy treated) samples.
(XLSX)

S6 Table Expression of ABCC3 in chemotherapy treated tumor samples. Represents the qPCR results of ABCC3expression in tumor breast tissue (chemotherapy treated) samples.
(XLSX)

S7 Table Effect of ABCC1 and ABCC3 knockdown on chemosensitivity. Represents the IC50 values of doxorubicin, mitoxantrone and methotrexate in cells stably expressing non-targeting shRNA or shABCC1 or shABCC3. IC50 values were determined with curvefit method using Graph-pad prism 5.
(PDF)

S8 Table Effect of ABCC3 knockdown on tumor inhibition rate. Represents the tumor inhibition rate of tumours formed in NOD SCID mice by MDA-MB-231 cells expressing NT or shABCC3 in the presence or absence of doxorubicin treatment.
(PDF)

## Abbreviations

DOX: Doxorubicin
MXR: Mitoxantrone
MTX: Methotrexate
5-FU: 5-Flurouracil
CT: Chemotherapy
NT: Non-targeting shRNA
ABC: ATP binding cassette
ct: Cycle threshold

## References

[1] Moses S, Shukla S, Mansoori IK. Retrospective and prospective study of stage presentation of carcinoma breast in tertiary health care centre, Indore. Journal of Evidence Based Medicene & Healthcare. 2015;2(17):2493–500.

[2] Gottesman MM. Mechanisms of cancer drug resistance. Annual review of medicine. 2002;53(1):615–27. 10.1146/annurev.med.53.082901.103929 11818492

[3] Cole SP, Deeley RG. Multidrug resistance mediated by the ATP-binding cassette transporter protein MRP. Bioessays. 1998;20(11):931–40. 9872059 10.1002/(SICI)1521-1878(199811)20:11<931::AID-BIES8>3.0.CO;2-J

[4] Saxena M, Stephens MA, Pathak H, Rangarajan A. Transcription factors that mediate epithelial-mesenchymal transition lead to multidrug resistance by upregulating ABC transporters. Cell death & disease. 2011;2:e179 10.1038/cddis.2011.61 21734725 PMC3199722

[5] Gottesman MM, Fojo T, Bates SE. Multidrug resistance in cancer: role of ATP-dependent transporters. Nat Rev Cancer. 2002;2(1):48–58. 11902585 10.1038/nrc706

[6] Vezmar M, Georges E. Reversal of MRP-mediated doxorubicin resistance with quinoline-based drugs. Biochemical Pharmacology. 2000;59(10):1245–52. 10.1016/S0006-2952(00)00270-7 10736425

[7] Hagmann W, Jesenofsky R, Faissner R, Guo C, Löhr JM. ATP-binding cassette C transporters in human pancreatic carcinoma cell lines. Pancreatology. 2008;9(1–2):136–44. 10.1159/000178884 19077464

[8] Burkhart CA, Watt F, Murray J, Pajic M, Prokvolit A, Xue C, et al Small-molecule multidrug resistance-associated protein 1 inhibitor reversan increases the therapeutic index of chemotherapy in mouse models of neuroblastoma. Cancer research. 2009;69(16):6573–80. 10.1158/0008-5472.CAN-09-1075 19654298 PMC2746061

[9] Zúñiga-García V, de Guadalupe Chávez-López M, Quintanar-Jurado V, Gabiño-López NB, Hernández-Gallegos E, Soriano-Rosas J, et al Differential Expression of Ion Channels and Transporters During Hepatocellular Carcinoma Development. Digestive diseases and sciences. 2015:1–11. 25842354 10.1007/s10620-015-3633-9

[10] Zhao Y, Lu H, Yan A, Yang Y, Meng Q, Sun L, et al ABCC3 as a marker for multidrug resistance in non-small cell lung cancer. Scientific Reports. 2013;3:3120 10.1038/srep03120 http://www.nature.com/articles/srep03120#supplementary-information. 24176985 PMC3814586

[11] Kuan C-T, Wakiya K, Herndon JE, Lipp ES, Pegram CN, Riggins GJ, et al MRP3: a molecular target for human glioblastoma multiforme immunotherapy. BMC Cancer. 2010;10:468- 10.1186/1471-2407-10-468 PMC2940806. 20809959 PMC2940806

[12] Filipits M, Malayeri R, Suchomel RW, Pohl G, Stranzl T, Dekan G, et al Expression of the multidrug resistance protein (MRP1) in breast cancer. Anticancer research. 1999;19(6B):5043–9. . 10697508

[13] Yamada A, Ishikawa T, Ota I, Kimura M, Shimizu D, Tanabe M, et al High expression of ATP-binding cassette transporter ABCC11 in breast tumors is associated with aggressive subtypes and low disease-free survival. Breast cancer research and treatment. 2013;137(3):773–82. 10.1007/s10549-012-2398-5 23288347 PMC3560367

[14] Partanen L, Staaf J, Tanner M, Tuominen VJ, Borg A, Isola J. Amplification and overexpression of the ABCC3 (MRP3) gene in primary breast cancer. Genes, chromosomes & cancer. 2012;51(9):832–40. 10.1002/gcc.21967 . 22585709

[15] Dey D, Saxena M, Paranjape AN, Krishnan V, Giraddi R, Kumar MV, et al Phenotypic and functional characterization of human mammary stem/progenitor cells in long term culture. PloS one. 2009;4(4):e5329 10.1371/journal.pone.0005329 19390630 PMC2669709

[16] Paranjape AN, Mandal T, Mukherjee G, Kumar MV, Sengupta K, Rangarajan A. Introduction of SV40ER and hTERT into mammospheres generates breast cancer cells with stem cell properties. Oncogene. 2012;31(15):1896–909. Epub 2011/08/30. 10.1038/onc.2011.378 onc2011378 [pii]. . 21874052

[17] Samardzija C, Luwor RB, Volchek M, Quinn MA, Findlay JK, Ahmed N. A critical role of Oct4A in mediating metastasis and disease-free survival in a mouse model of ovarian cancer. Molecular Cancer. 2015;14:152 10.1186/s12943-015-0417-y PMC4531496. 26260289 PMC4531496

[18] Paranjape AN, Balaji SA, Mandal T, Krushik EV, Nagaraj P, Mukherjee G, et al Bmi1 regulates self-renewal and epithelial to mesenchymal transition in breast cancer cells through Nanog. BMC Cancer. 2014;14:785 10.1186/1471-2407-14-785 25348805 PMC4223733

[19] Liu S, Luo X, Li D, Zhang J, Qiu D, Liu W, et al Tumor inhibition and improved immunity in mice treated with flavone from Cirsium japonicum DC. International Immunopharmacology. 2006;6(9):1387–93. 10.1016/j.intimp.2006.02.002 16846832

[20] Atalay C, Demirkazik A, Gunduz U. Role of ABCB1 and ABCC1 gene induction on survival in locally advanced breast cancer. Journal of chemotherapy. 2008;20(6):734–9. 10.1179/joc.2008.20.6.734 . 19129072

[21] Fletcher JI, Haber M, Henderson MJ, Norris MD. ABC transporters in cancer: more than just drug efflux pumps. Nat Rev Cancer. 2010;10(2):147–56. 10.1038/nrc2789 20075923

[22] El Azreq M-A, Naci D, Aoudjit F. Collagen/β1 integrin signaling up-regulates the ABCC1/MRP-1 transporter in an ERK/MAPK-dependent manner. Molecular Biology of the Cell. 2012;23(17):3473–84. 10.1091/mbc.E12-02-0132 PMC3431945. 22787275 PMC3431945

[23] Forster S, Thumser AE, Hood SR, Plant N. Characterization of rhodamine-123 as a tracer dye for use in in vitro drug transport assays. PloS one. 2012;7(3). 10.1371/journal.pone.0033253 PMC3314654 22470447

[24] Zhao M, Zhang Y, Zhang H, Wang S, Zhang M, Chen X, et al Hypoxia-induced cell stemness leads to drug resistance and poor prognosis in lung adenocarcinoma. Lung cancer. 2015;87(2):98–106. 10.1016/j.lungcan.2014.11.017 . 25512094

[25] Al-Hajj M, Wicha MS, Benito-Hernandez A, Morrison SJ, Clarke MF. Prospective identification of tumorigenic breast cancer cells. Proceedings of the National Academy of Sciences of the United States of America. 2003;100(7):3983–8. 10.1073/pnas.0530291100 12629218 PMC153034

[26] Tsou S-H, Chen T-M, Hsiao H-T, Chen Y-H. A Critical Dose of Doxorubicin Is Required to Alter the Gene Expression Profiles in MCF-7 Cells Acquiring Multidrug Resistance. PloS one. 2015;10(1):e0116747 10.1371/journal.pone.0116747 25635866 PMC4312059

[27] Munoz M, Henderson M, Haber M, Norris M. Role of the MRP1/ABCC1 multidrug transporter protein in cancer. IUBMB life. 2007;59(12):752 18085475 10.1080/15216540701736285

[28] Zhao Y, Lu H, Yan A, Yang Y, Meng Q, Sun L, et al ABCC3 as a marker for multidrug resistance in non-small cell lung cancer. Sci Rep. 2013;3 10.1038/srep03120 http://www.nature.com/srep/2013/131101/srep03120/abs/srep03120.html#supplementary-information. PMC3814586 24176985

[29] Gillet J-P, Efferth T, Remacle J. Chemotherapy-induced resistance by ATP-binding cassette transporter genes. Biochimica et Biophysica Acta (BBA)—Reviews on Cancer. 2007;1775(2):237–62. 10.1016/j.bbcan.2007.05.002 17572300

[30] Bram EE, Stark M, Raz S, Assaraf YG. Chemotherapeutic Drug-Induced ABCG2 Promoter Demethylation as a Novel Mechanism of Acquired Multidrug Resistance. Neoplasia (New York, NY). 2009;11(12):1359–70. PMC2794517. 10.1593/neo.91314 PMC2794517 20019844

[31] Li Q-Q, Xu J-D, Wang W-J, Cao X-X, Chen Q, Tang F, et al Twist1-mediated adriamycin-induced epithelial-mesenchymal transition relates to multidrug resistance and invasive potential in breast cancer cells. Clinical Cancer Research. 2009;15(8):2657–65. 10.1158/1078-0432.CCR-08-2372 19336515

[32] El-Osta A, Kantharidis P, Zalcberg JR, Wolffe AP. Precipitous Release of Methyl-CpG Binding Protein 2 and Histone Deacetylase 1 from the Methylated Human Multidrug Resistance Gene (MDR1) on Activation. Molecular and Cellular Biology. 2002;22(6):1844–57. 10.1128/MCB.22.6.1844-1857.2002 PMC135609. 11865062 PMC135609

[33] Kusaba H, Nakayama M, Harada T, Nomoto M, Kohno K, Kuwano M, et al Association of 5′ CpG demethylation and altered chromatin structure in the promoter region with transcriptional activation of the multidrug resistance 1 gene in human cancer cells. European Journal of Biochemistry. 1999;262(3):924–32. 10.1046/j.1432-1327.1999.00469.x 10411657

[34] Calcagno AM, Fostel JM, To KKW, Salcido CD, Martin SE, Chewning KJ, et al Single-step doxorubicin-selected cancer cells overexpress the ABCG2 drug transporter through epigenetic changes. British Journal of Cancer. 2008;98(9):1515–24. 10.1038/sj.bjc.6604334 PMC2386965. 18382425 PMC2386965

[35] To KKW, Polgar O, Huff LM, Morisaki K, Bates SE. Histone Modifications at the ABCG2 Promoter following Treatment with Histone Deacetylase Inhibitor Mirror Those in Multidrug-Resistant Cells. Molecular Cancer Research. 2008;6(1):151–64. 10.1158/1541-7786.MCR-07-0175 PMC3306834. 18234970 PMC3306834

[36] Henderson MJ, Haber M, Porro A, Munoz MA, Iraci N, Xue C, et al ABCC multidrug transporters in childhood neuroblastoma: clinical and biological effects independent of cytotoxic drug efflux. Journal of the National Cancer Institute. 2011;103(16):1236–51. 10.1093/jnci/djr256 21799180 PMC3156802

[37] Susanto J, Lin Y-H, Chen Y-N, Shen C-R, Yan Y-T, Tsai S-T, et al Porphyrin Homeostasis Maintained by ABCG2 Regulates Self-Renewal of Embryonic Stem Cells. PloS one. 2008;3(12):e4023 10.1371/journal.pone.0004023 19107196 PMC2602981

[38] Chen Z, Liu F, Ren Q, Zhao Q, Ren H, Lu S, et al Suppression of ABCG2 inhibits cancer cell proliferation. International Journal of Cancer. 2010;126(4):841–51. 10.1002/ijc.24796 19642144

[39] Kuo MT. Redox Regulation of Multidrug Resistance in Cancer Chemotherapy: Molecular Mechanisms and Therapeutic Opportunities. Antioxidants & Redox Signaling. 2009;11(1):99–133. 10.1089/ars.2008.2095 PMC2577715. 18699730 PMC2577715

[40] Wijnholds J, Evers R, van Leusden MR, Mol CA, Zaman GJ, Mayer U, et al Increased sensitivity to anticancer drugs and decreased inflammatory response in mice lacking the multidrug resistance-associated protein. Nature medicine. 1997;3(11):1275–9. 9359705 10.1038/nm1197-1275

