# Supplementary material for: Role of the Drug Transporter ABCC3 in Breast Cancer Chemoresistance
Source: PLoS One. 2016 May 12;11(5):e0155013. doi: 10.1371/journal.pone.0155013 (PMC4865144; doi:10.1371/journal.pone.0155013)
Supplement: S1 Fig — This data file contains a list of the primers and sequences for the same, that were used in Real time PCR experiments. (PDF) [file pone.0155013.s001.pdf]

Gene name	Primer sequence
ABCC1-FW	5’-AGCCGGTGAAGGTTGTGTAC -3’
ABCC1-RV	5’-TGACGAAGCAGATGTGGAAG -3’
ABCC3-FW	5’-CCTTTGCCAACTTTCTCTGC -3’
ABCC3-RV	5’-AGGGCACTCAGCTGTCTCAT -3’
BMI1-FW	5’- CCAGGGCTTTTCAAAAATGA -3’
BMI1-RV	5’-CCGATCCAATCTGTTCTGGT -3’
Nanog-FW	5’- CCTCCTCCATGGATCTGCTTATTCA -3’
Nanog-RV	5’-CAGGTCTTCACCTGTTTGTAG -3’
Oct-4-FW	5’-CGACCATCTGCCGCTTTGAG -3’
Oct-4-RW	5’-CCCCCTGTCCCCCATTCCTA -3’
β2M-FW	5’-CCTGAATTGCTATGTGTCTGGG-3’
β2M-RW	5’-TGATGCTGCTTACATGTCTCGA -3’
GAPDH-FW	5’-CCACCCATGGCAAATTCCATGGCA-3’
GAPDH-RV	5’-TCTAGACGGCAGGTCAGGTCCACC-3’

S1 Fig. Primers for Real time PCR experiments: The sequence of the primers used in the study.
