# Supplementary material for: Role of the Drug Transporter ABCC3 in Breast Cancer Chemoresistance
Source: PLoS One. 2016 May 12;11(5):e0155013. doi: 10.1371/journal.pone.0155013 (PMC4865144; doi:10.1371/journal.pone.0155013)
Supplement: S2 Fig — FACS plots representing the drug retention in MD-AMB-231 and BT-474 cells (S2B and S2D) transiently transfected with empty vector or ABCC1 (OE) or ABCC3 (OE); n = 3. (PDF) [file pone.0155013.s002.pdf]

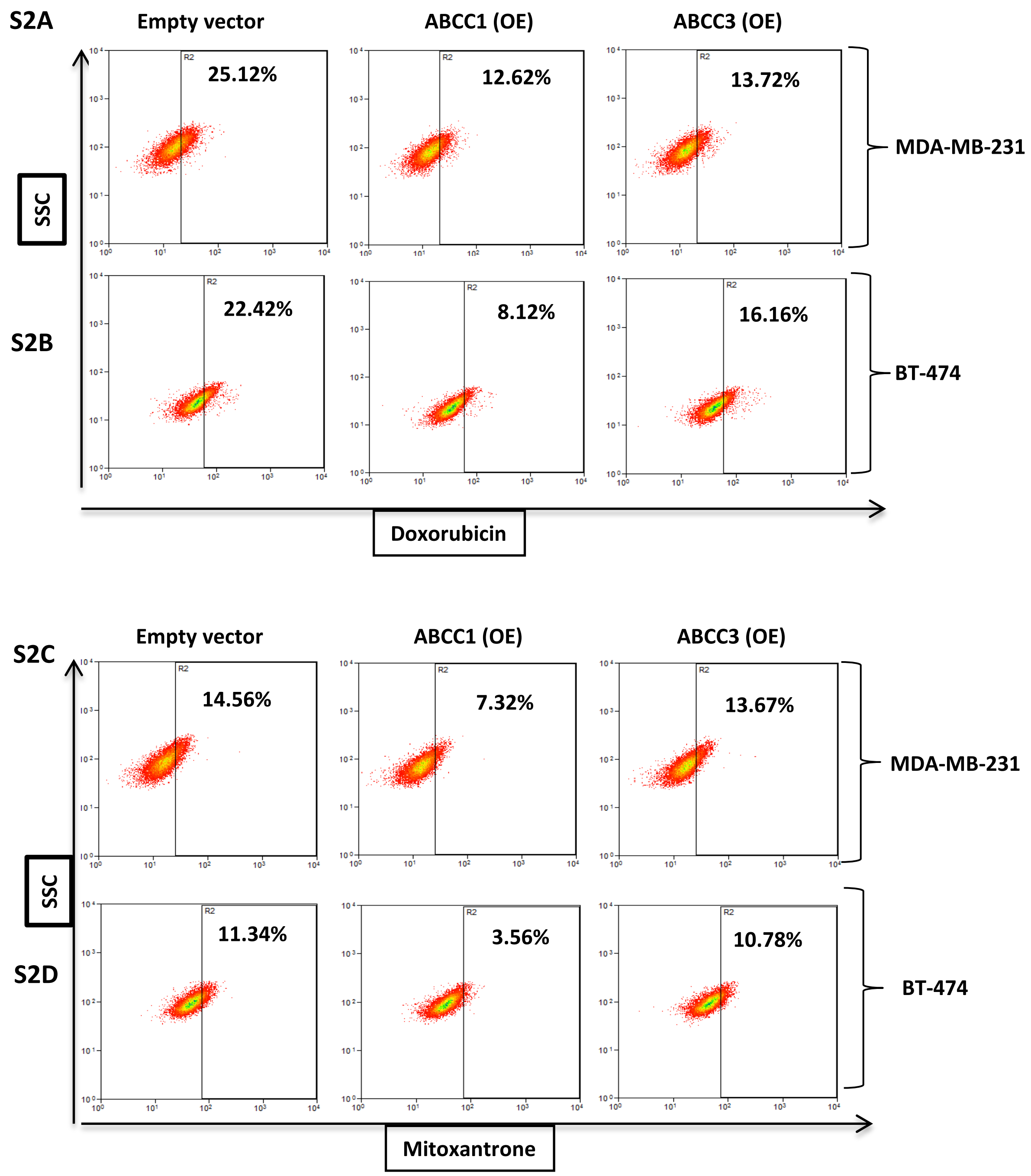

S2 Fig. Effect of ABCC3 overexpression on chemotherapeutic drug retention: FACS plots representing the drug retention in MD-AMB-231 (S2A and S2C) and BT-474 cells (S2B and S2D) transiently transfected with empty vector or ABCC1 (OE) or ABCC3 (OE). n=3.
