# Supplementary material for: Role of the Drug Transporter ABCC3 in Breast Cancer Chemoresistance
Source: PLoS One. 2016 May 12;11(5):e0155013. doi: 10.1371/journal.pone.0155013 (PMC4865144; doi:10.1371/journal.pone.0155013)
Supplement: S3 Fig — FACS plots represents rhodamine-123 retention in MK-571 treated breast cancer cells (S3A), doxorubicin retention in MK-571 treated breast cancer cells (S3B) and in primary breast cancer cells (S3C). n = 3. (PDF) [file pone.0155013.s003.pdf]

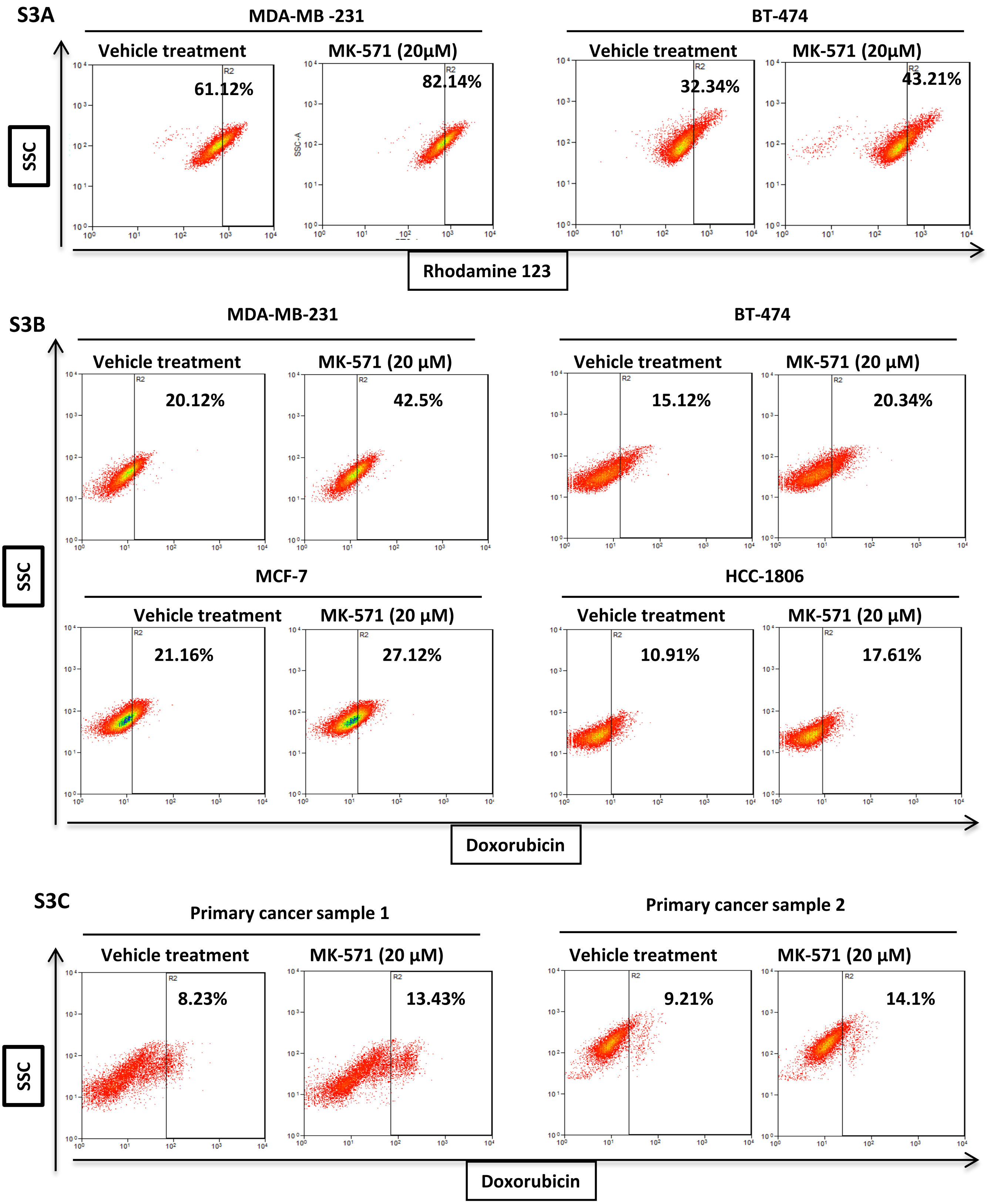

S3 Fig. Effect of MK-571 on Rhodamine and Doxorubicin retention in breast cancer cells: FACS plots represents rhodamine-123 retention in MK-571 treated breast cancer cells (S3A), doxorubicin retention in MK-571 treated breast cancer cells (S3B) and in primary breast cancer cells (S3C). n=3.
