# Supplementary material for: Role of the Drug Transporter ABCC3 in Breast Cancer Chemoresistance
Source: PLoS One. 2016 May 12;11(5):e0155013. doi: 10.1371/journal.pone.0155013 (PMC4865144; doi:10.1371/journal.pone.0155013)
Supplement: S4 Fig — FACS plots shows retention of increasing concentrations of doxorubicin in MDA-MB-231 cells expressing NT or shABCC1 or shABCC3.; n = 3. (PDF) [file pone.0155013.s004.pdf]

S4A

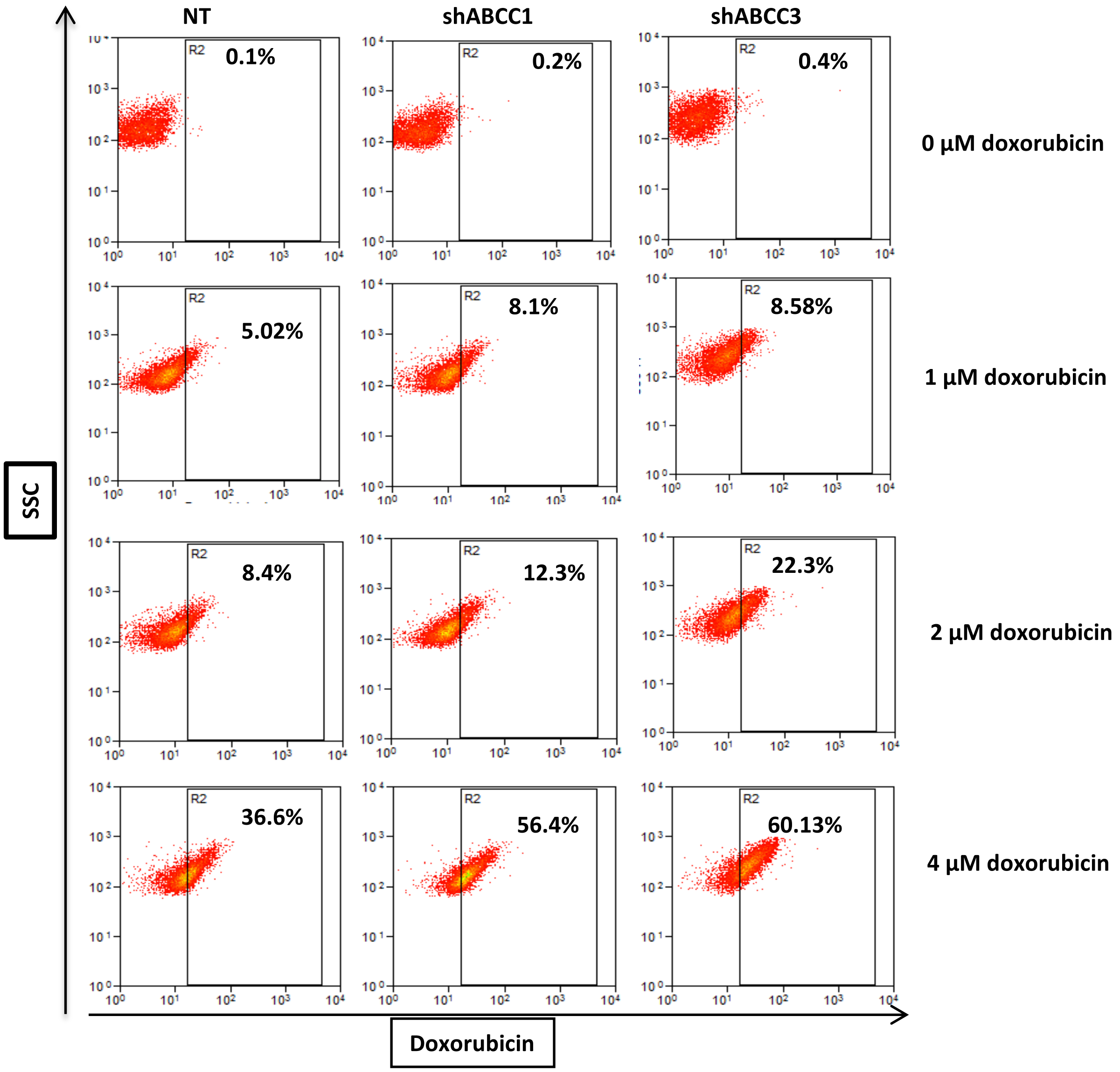

S4A Fig. Effect of ABCC3 knockdown on doxorubicin retention: FACS plots shows retention of increasing concentrations of doxorubicin in MDA-MB-231 cells expressing NT or shABCC1 or shABCC3. n=3.
