# Supplementary material for: Role of the Drug Transporter ABCC3 in Breast Cancer Chemoresistance
Source: PLoS One. 2016 May 12;11(5):e0155013. doi: 10.1371/journal.pone.0155013 (PMC4865144; doi:10.1371/journal.pone.0155013)
Supplement: S5 Fig — The FACS plots representing the percentage of Annexin-V-PE-cy5 staining in MDA-MB-231 expressing NT, shABCC1 and shABCC3 cells treated with doxorubicin or methotrexate for 20 hours. (PDF) [file pone.0155013.s005.pdf]

S5A

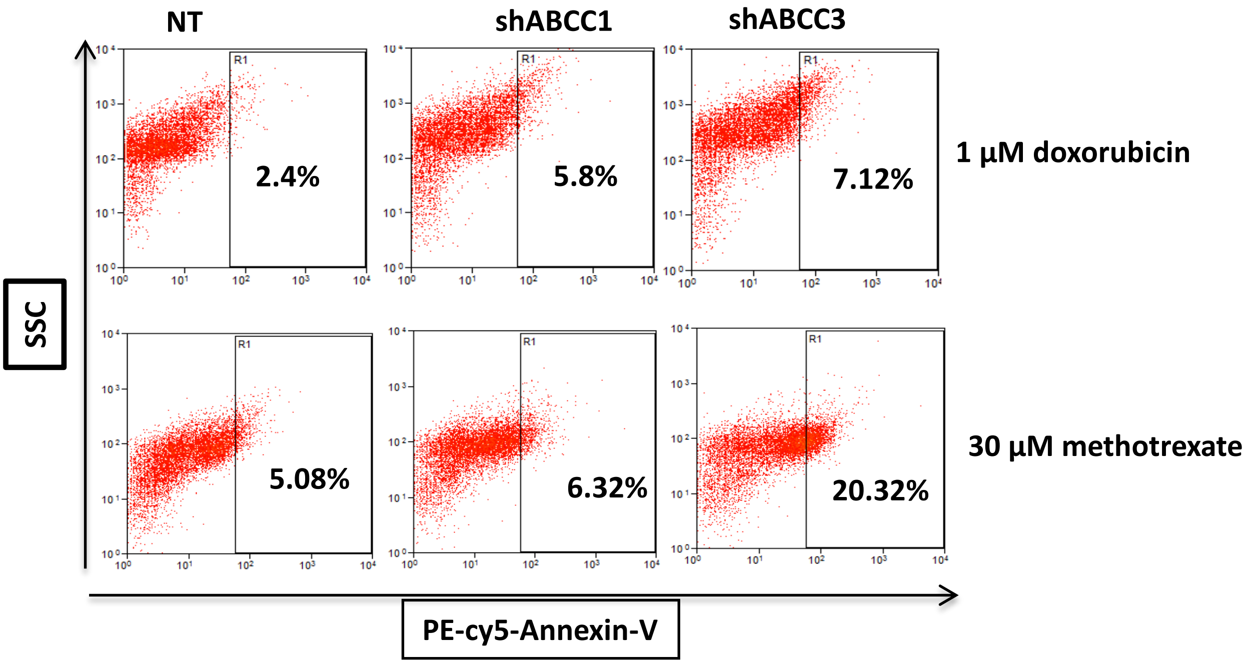

S5A Fig. Effect of ABCC3 knockdown on drug induced apoptosis: The FACS plots representing the percentage of Annexin-V-PE-cy5 staining in MDA-MB-231 expressing NT, shABCC1 and shABCC3 cells treated with doxorubicin or methotrexate for 20 hours. n=3.
