# Supplementary material for: Role of the Drug Transporter ABCC3 in Breast Cancer Chemoresistance
Source: PLoS One. 2016 May 12;11(5):e0155013. doi: 10.1371/journal.pone.0155013 (PMC4865144; doi:10.1371/journal.pone.0155013)
Supplement: S7 Table — Represents the IC50 values of doxorubicin, mitoxantrone and methotrexate in cells stably expressing non-targeting shRNA or shABCC1 or shABCC3. IC50 values were determined with curvefit method using Graph-pad prism 5. (PDF) [file pone.0155013.s012.pdf]

S7 Table. Effect of ABCC3 knockdown on chemosensitivity

Cell lines	IC_{50} values		
MDA-MB-231	Doxorubicin (µM)	Mitoxanthrone (µM)	Methotrexate (µM)
NT	0.986 ± 0.08142	0.816 ± 0.092	24.12 ± 1.042
shABCC1	0.601 ± 0.05932	0.365 ± 0.0732	21.34 ± 0.936
shABCC3	0.41 ± 0.0843	0.625 ± 0.094	13.35 ± 0.731

S7 Table. Represents the IC_{50} values of doxorubicin, mitoxantrone and methotrexate in cells stably expressing non-targeting shRNA or shABCC1 or shABCC3. IC_{50} values were determined with curve-fit method using Graph-pad prism 5.
