# Supplementary material for: Role of the Drug Transporter ABCC3 in Breast Cancer Chemoresistance
Source: PLoS One. 2016 May 12;11(5):e0155013. doi: 10.1371/journal.pone.0155013 (PMC4865144; doi:10.1371/journal.pone.0155013)
Supplement: S8 Table — Represents the tumor inhibition rate of tumours formed in NOD SCID mice by MDA-MB-231 cells expressing NT or shABCC3 in the presence or absence of doxorubicin treatment. (PDF) [file pone.0155013.s013.pdf]

S8 Table. Effect of ABCC3 knockdown on tumor inhibition rate

Tumors	Inhibition Rate (%)
NT	-
NT+DOX	62.13
shABCC3	48.89
shABCC3+DOX	77.58

S8 Table. Represents the tumor inhibition rate of tumours formed in NOD SCID mice by MDA-MB-231 cells expressing NT or shABCC3 in the presence or absence of doxorubicin treatment. (PDF)
